# Tunable Fluorescence and Afterglow in Organic Crystals for Temperature Sensing

**DOI:** 10.1021/acs.jpclett.2c00168

**Published:** 2022-02-21

**Authors:** Jian-Xin Wang, Ling-Ya Peng, Zheng-Fei Liu, Xin Zhu, Li-Ya Niu, Ganglong Cui, Qing-Zheng Yang

**Affiliations:** † Key Laboratory of Radiopharmaceuticals, Ministry of Education, College of Chemistry, Beijing Normal University, Beijing 100875, P. R. China; ‡ Key Laboratory of Theoretical and Computational Photochemistry, Ministry of Education, College of Chemistry, Beijing Normal University, Beijing 100875, P. R. China; § Advanced Membranes and Porous Materials Center, Division of Physical Science and Engineering, King Abdullah University of Science and Technology, Thuwal 23955-6900, Kingdom of Saudi Arabia

## Abstract

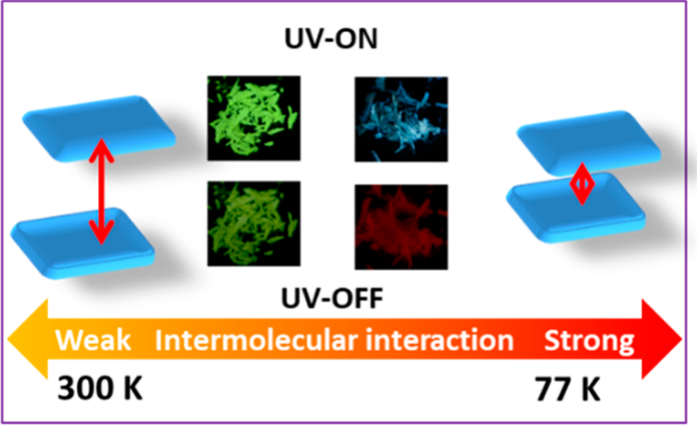

The modulation of the properties of emission from multiple emission states in a single-component organic luminescent material is highly desirable in data anticounterfeiting, information storage, and bioapplications. Here, a single-component luminescent organic crystal of difluoroboron diphenyl β-diketonate with controllable multiple emission colors is successfully reported. The temperature-dependent luminescence experiments supported by high-level theoretical calculations demonstrate that the ratio of the fluorescence between the monomer and excimer and the phosphorescence maxima of the excimer can be effectively regulated. In addition, the temperature-dependent fluorescence and afterglow dual-emission color changes provide a new strategy for the design of highly accurate double-checked temperature sensors.

Solid organic luminescent materials with multiple emission colors have attracted a great deal of attention due to their fascinating molecular compositions, rich photophysical properties, and enormous potentials in optoelectronic and biomedical applications.^1−14^ Multicolor emission systems prepared from composite materials have been widely investigated. However, their preparation is very complex, and the different materials used in preparing them are poorly compatible with each other, which seriously affects their performance in practical applications.^15−22^ As a result, there is an urgent need to develop single-component metal-free materials with multicolor emissions, excellent durability, and stability. In reported single-component materials, multicolor emissions are usually achieved via manipulating intermolecular packing modes during the synthesis of the materials and growth of crystals.^16,18,23,24^ In contrast, it is still difficult to obtain stimulus-responsive single-component materials with multicolor emissions especially those with more than two emission colors.^25−28^

The luminescence mechanism of organic materials in solid states,^24,29−37^ especially single-component organic crystals with multiple emissions, is very complicated.^23,27,28,38^ Small variations in molecular conformation, intermolecular packing mode, sample morphology, or even surroundings of materials could affect their luminescence properties.^13,39^ Thus, it is very important to explore the luminescence mechanism of multiple-emission materials for their rapid development. The excimer that is formed between a ground-state molecule and an excited-state one is a common luminescence phenomenon found in many chromophores.^40^ As the excimer-based sensors precisely probe analytes using the luminescence changes during the monomer–dimer conversion,^41−43^ the dynamic formation and dissociation of the excimer may be used as one of the ingenious mechanisms for designing single-component multicolor emitters. Difluoroboron β-diketonate derivatives are some of the potential materials for studying stimulus-responsive luminescence systems with multiple emissions due to their modifiable molecular conformation and intermolecular interaction.^20,26,44−50^ In addition, the n−π electronic transition of difluoroboron β-diketonate enables room-temperature phosphorescence and afterglow emission through appropriate regulation of the π system according to El-Sayed’s rule.^17,51−53^ These characteristics fully provide the prerequisite for the establishment of a multiple-emission system in a single-component luminescent material.

Here, we present a single-component organic molecular crystal, difluoroboron diphenyl β-diketonate (**M1**), with temperature-modulated fluorescence and afterglow emissions. The experimental and theoretical calculation results demonstrate that a temperature-dependent fluorescence color change from yellow-green (540 nm, 300 K) to blue (470 nm, 77 K) and an afterglow color change from yellow (560 nm, 300 K) to red (650 nm, 77 K) are due to temperature-modulated intermolecular interactions. At 300 K, the emission of the excimer dominates the whole spectra, but at low temperatures, the formation of the excimer is inhibited and monomer fluorescence increases gradually. Moreover, enhanced intermolecular interaction shifts excimer fluorescence from 540 to 560 nm and phosphorescence from 560 to 650 nm. Therefore, yellow-green excimer fluorescence and yellow excimer afterglow are observed at 300 K, whereas blue monomer fluorescence and red excimer afterglow are observed at 77 K. The ratio of fluorescence between the monomer and excimer correlates linearly with temperature, while phosphorescence maxima have a good linear relationship with temperature. The synergistic temperature response of fluorescence and afterglow of the **M1** crystal makes it highly sensitive and accurate in sensing temperature.

**M1** crystals exhibit yellow fluorescence at 540 nm with a small shoulder at 470 nm; they also show a phosphorescence band at 560 nm with a very weak shoulder at 650 nm at room temperature (Figure 1a and Figure S1). The concentration-dependent fluorescence spectra of **M1** in a chloroform solution were recorded at room temperature (Figure 1b). A new unstructured broadened emission band at ∼540 nm appeared and gradually increased as the concentration of **M1** increased from 1 μM to 200 mM. This is in line with the emission characteristics of the excimer. The emission band of the monomer at 400 nm gradually red-shifted to 470 nm, which could probably be caused by the enhanced intermolecular interaction at high concentrations. The peak positions of the monomer and excimer fluorescence are basically consistent with the peak in the crystal. Therefore, the emission at 540 and 470 nm in the crystal is assigned to the fluorescence of the excimer and monomer, respectively.

**Figure 1:**
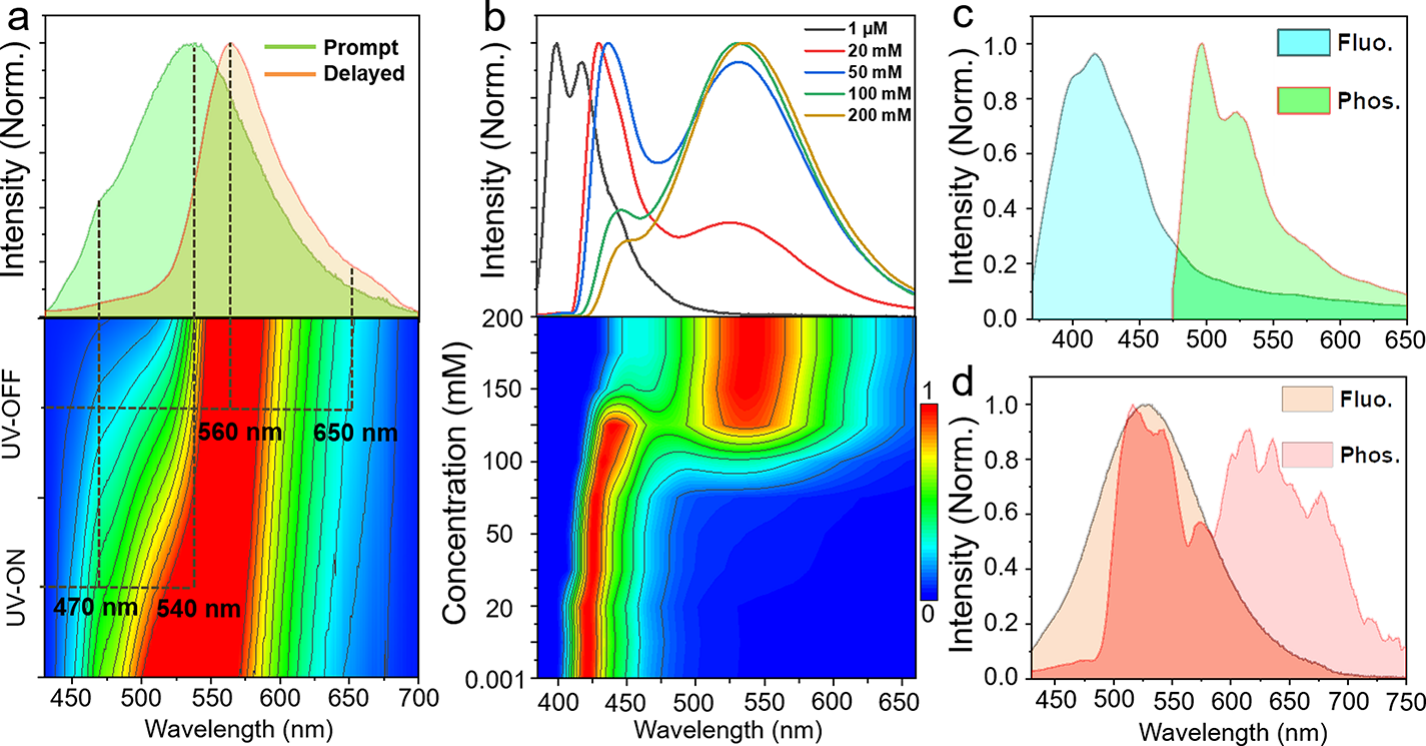
(a) Fluorescence (green line) and phosphorescence (red line, delayed 1 ms) spectra of **M1** crystals. (b) Concentration-dependent emission spectra and corresponding emission mapping of **M1** in chloroform solutions. Fluorescence (at room temperature) and phosphorescence (delayed 1 ms, at 77 K) spectra of (c) 1 wt % and (d) 50 wt % **M1** doped in PMMA films.

To determine the attribution of these two phosphorescence bands at 560 and 650 nm, the emission spectra of **M1** in a poly(methyl methacrylate) (PMMA) film (Figure 1c,d) and a chloroform solution with different concentrations were recorded (Figures S2 and S3). At room temperature, only the monomer’s fluorescence at 400 nm was observed (1 wt % in PMMA or 1 μM in a chloroform solution). The delayed emission band at around 525 nm could be observed only at 77 K, which is assigned to the phosphorescence of the monomer. Interestingly, a new broad phosphorescence band at 650 nm was detected at 77 K as the concentration of **M1** increased to 50 wt % in PMMA (Figure 1d) or 200 mM (Figure S3) in a chloroform solution, which is assigned to the excimer-correlated phosphorescence.

The most important finding of this work is the temperature-dependent fluorescence and afterglow color changes in **M1** crystals. The fluorescence intensity at 470 nm subtly increased, while the fluorescence at 540 nm increased and gradually red-shifted to 560 nm as the temperature decreased from 300 to 200 K, due to the stabilizing effect of cooling on the excimer complex. The further increase in the fluorescence at 470 nm and the decrease in the fluorescence at 560 nm resulted from the suppression of the excimer formation process (Figure 2a). At the same time, the phosphorescence at 560 nm gradually red-shifts to 650 nm with the intensity increasing first and then decreasing. This is also consistent with the temperature stabilization and suppression effect on the excimer formation process (Figure 2b).

**Figure 2:**
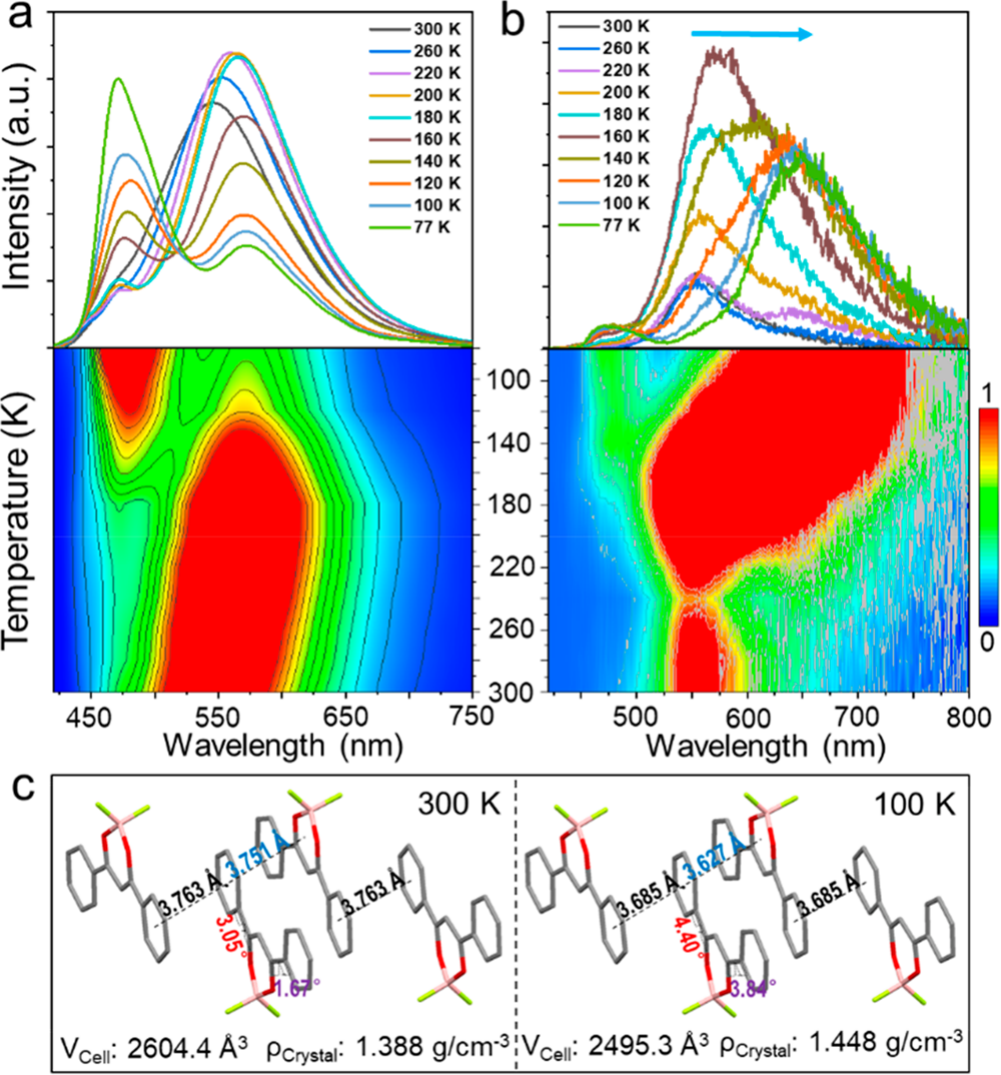
Temperature-dependent (a) fluorescence and (b) phosphorescence (delayed 1 ms) spectra of **M1** crystals and corresponding emission mappings. (c) Selected crystal patterns, volumes, and densities of **M1** crystals at different temperatures.

The red-shift of the excimer fluorescence and phosphorescence spectra was further investigated by the crystal structures of **M1** at different temperatures (Figure 2c and Table S1). It is noteworthy that there was no significant overlap between the dioxaborine ring and the adjacent molecules. The antiparallel alignment of neighboring molecules indicated moderate intermolecular interactions at room temperature. At low temperatures, the centroid–centroid distance of the two phenyls between two adjacent molecules gradually decreased from 3.763 to 3.685 Å while the distance between the difluoroboron β-diketonate moiety and the phenyl group from the molecule in the vicinity decreased from 3.751 to 3.627 Å. Macroscopically, the volume of the recorded crystal decreases from 2604.4 to 2495.3 Å^3^, which corresponded to an increase in the crystal density from 1.388 to 1.448 g/cm^3^ at low temperatures. Therefore, the enhanced intermolecular interaction was observed at low temperatures, which is the main contribution of the red-shift of the fluorescence and phosphorescence.

The temperature-dependent fluorescence and afterglow emissions are rationalized by theoretical calculations. The S_0_ structures are optimized with the B3LYP method, while the S_1_ and T_1_ structures are determined with the TD-B3LYP method (see the Supporting Information for details).^54−58^ The polarizable continuum medium (PCM) model is also used to consider solvent effects.^59^ The energies of all of the optimized structures are further refined at the CASPT2 and CASPT2/PCM levels.^60,61^ The vertical fluorescence and phosphorescence emission energies in chloroform solutions at 408 and 543 nm are consistent with the experimental data at 400 and 525 nm, respectively. These results show that the fluorescence and phosphorescence stem from the S_1_ and T_1_ states (Table S4).

**Figure 3:**
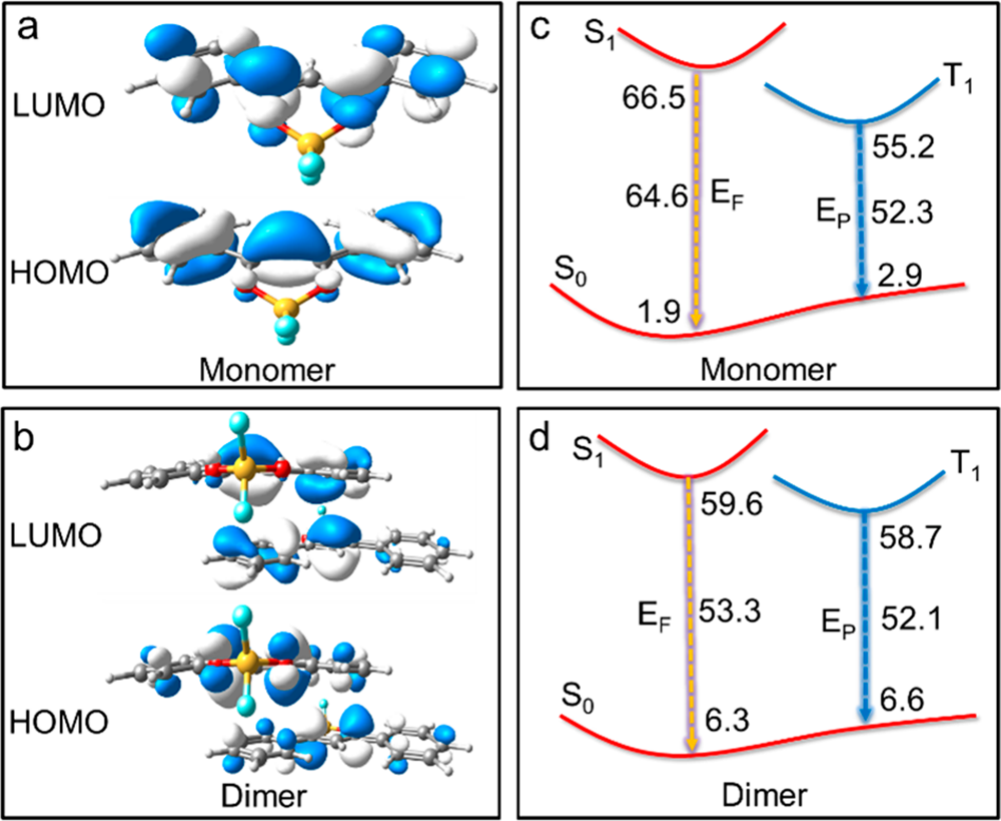
Frontier molecular orbitals at the S_0_ minima of (a) monomer and (b) dimer models of **M1** crystals. Proposed luminescence models of the (c) monomer and (d) dimer. The S_0_, S_1_, and T_1_ energies (kilocalories per mole) are calculated at the QM(CASPT2)/MM level for optimized T_1_ and S_1_ structures. The vertical emission energies are the energy differences between S_1_ and S_0_ (*E*_F_) or T_1_ and S_0_ (*E*_P_) calculated above.

The optimized S_0_, S_1_, and T_1_ structures of the **M1** monomer and dimer in the crystal are also determined at the QM(CASPT2)/MM level (Figure 3).^62,63^ The vertical fluorescence emission energy of the monomer at 443 nm matches well with that at 470 nm measured by the experiment. The fluorescence and phosphorescence emissions of the dimer at 536 and 548 nm are in accordance with the experimental emissions at 540 and 560 nm, respectively, in the crystal. The red-shift of the fluorescence and phosphorescence of the **M1** dimer at low temperatures is also confirmed by the calculation results. As the centroid–centroid intermolecular distance within one dimer is decreased, the calculated fluorescence and phosphorescence of the dimer are red-shifted to 552 and 627 nm. The red-shifts of the fluorescence and phosphorescence spectra at shorter intermolecular distance from the theoretical simulation are in good agreement with the results of temperature-dependent spectroscopy experiments. This further confirms that the dual temperature-dependent characteristics of fluorescence and afterglow emission of **M1** crystals are caused by the change in intermolecular interactions at different temperatures.

To date, most of the traditional temperature-responsive luminescent materials use the single emission intensity or lifetime changes to monitor temperature fluctuation, which is also influenced by the surroundings. To improve the accuracy and sensitivity of thermometers, ratiometric temperature sensors are a good alternative, which can accurately measure temperature by the change in the ratio of the intensity or lifetime of two or more emissions. Therefore, the temperature-dependent fluorescence and afterglow emission color changes of **M1** crystals provide a new strategy for designing double-checked temperature sensors with significantly improved sensitivity and accuracy. The fluorescence ratio between the monomer and excimer linearly correlates with temperature (Figure 4a), while the phosphorescence maximum has a good linear relationship with temperature (Figure 4b). The synergistic temperature response of the fluorescence and afterglow luminescence of the **M1** crystal endow it with high sensitivity and accuracy in sensing temperature. In addition, the temperature-dependent fluorescence and afterglow color change can be clearly and directly observed by the naked eye or with a camera or by comparison with the temperature-dependent CIE chromaticity diagram (Figure 4c,d).

**Figure 4:**
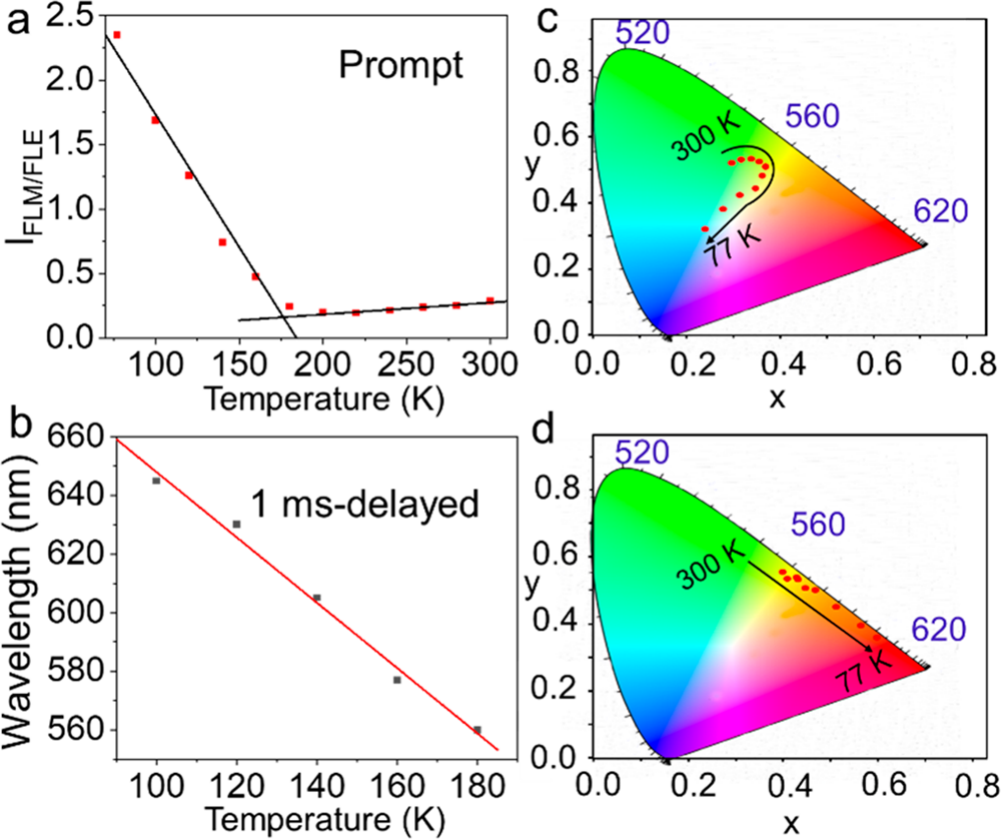
(a) Linear relationship between *I*_FLM/FLE_ and temperature (*I*_FLM/FLE_ is the ratio of the fluorescence intensities between the **M1** monomer and excimer). (b) Linear relationship between the phosphorescence maxima of **M1** crystals and temperature. (c and d) CIE coordinates transformed from panels a and b, respectively, of Figure 2.

In summary, a single-component organic crystal difluoroboron 1,3-diphenyl β-diketonate (**M1**) with stimulus-responsive multiple emissions was successfully constructed and investigated. The temperature-dependent spectroscopy experiments supported by high-level theoretical calculations demonstrate that the temperature-dependent fluorescence color of **M1** crystals changes from yellow-green (540 nm, 300 K) to blue (470 nm, 77 K) and the afterglow color changes from yellow (560 nm, 300 K) to red (650 nm, 77 K) due to the temperature-modulated intermolecular interactions. The ratio of the fluorescence between the monomer and excimer correlates linearly with temperature, while the phosphorescence maxima have a good linear relationship with temperature. The synergistic temperature response of the fluorescence and afterglow emissions of **M1** crystals make it sensitive and accurate in sensing temperature.

## Supporting Information Available

Experimental methods, theoretical modeling framework, and characterization results (PDF)

## References

[ref1] Wang J.-X.; Gutiérrez-Arzaluz L.; Wang X.; Almalki M.; Yin J.; Czaban-Jóźwiak J.; Shekhah O.; Zhang Y.; Bakr O. M.; Eddaoudi M.; et al. Nearly 100% Energy Transfer at the Interface of Metal-Organic Frameworks for X-ray Imaging Scintillators. Matter. 2022, 5, 253–265. 10.1016/j.matt.2021.11.012.

[ref2] Wang J.-X.; Wang X.; Yin J.; Gutiérrez-Arzaluz L.; He T.; Chen C.; Han Y.; Zhang Y.; Bakr O. M.; Eddaoudi M.; et al. Perovskite-Nanosheet Sensitizer for Highly Efficient Organic X-ray Imaging Scintillator. ACS. Energy. Lett. 2022, 7, 10–16. 10.1021/acsenergylett.1c02173.

[ref3] Bhaumik S. K.; Banerjee S. Tunable Multi-Color Luminescence from a Self-Assembled Cyanostilbene and Cucurbit[7]uril in Aqueous Media. Chem. Commun. 2020, 56, 655–658. 10.1039/C9CC09277C. 31840149

[ref4] Li M.; Zhang Y.; Ren X.; Niu W.; Yuan Q.; Cao K.; Zhang J.; Gao X.; Su D. Activatable Fluorogenic Probe for Accurate Imaging of Ulcerative Colitis Hypoxia in Vivo. Chem. Commun. 2022, 58, 819–822. 10.1039/D1CC06577G. 34928281

[ref5] Matsuo K.; Thayyil S.; Kawaguchi M.; Nakagawa H.; Tamaoki N. A Visible Light-Controllable Rho Kinase Inhibitor based on a Photochromic Phenylazothiazole. Chem. Commun. 2021, 57, 12500–12503. 10.1039/D1CC04905D. 34751279

[ref6] Smith K. T.; Ramsperger C. A.; Hunter K. E.; Zuehlsdorff T. J.; Stylianou K. C. Colorimetric Detection of Acidic Pesticides in Water. Chem. Commun. 2022, 58, 953–956. 10.1039/D1CC06213A. 34940765

[ref7] Gavara R.; Gago S.; Jordao N.; Pina F. 4’-Carboxy-7-Hydroxyflavylium. A Multistate System Involving Twelve Species Reversibly Interconverted by pH and Light Stimuli. J. Phys. Chem. A 2014, 118, 4723–4731. 10.1021/jp503790z. 24892692

[ref8] Mukherjee S.; Sahoo A.; Deb S.; Baitalik S. Light and Cation-Driven Optical Switch based on a Stilbene-Appended Terpyridine System for the Design of Molecular-Scale Logic Devices. J. Phys. Chem. A 2021, 125, 8261–8273. 10.1021/acs.jpca.1c06524. 34506718

[ref9] Morris W. A.; Butler T.; Kolpaczynska M.; Fraser C. L. Stimuli Responsive Furan and Thiophene Substituted Difluoroboron beta-Diketonate Materials. Mater. Chem. Front. 2017, 1, 158–166. 10.1039/C6QM00008H. 28239477 PMC5321547

[ref10] Butler T.; Wang F.; Sabat M.; Fraser C. L. Controlling Solid-State Optical Properties of Stimuli Responsive Dimethylamino-Substituted Dibenzoylmethane Materials. Mater. Chem. Front. 2017, 1, 1804–1817. 10.1039/C7QM00157F.

[ref11] Li B.; Lin C.; Lu C.; Zhang J.; He T.; Qiu H.; Yin S. A Rapid and Reversible Thermochromic Supramolecular Polymer Hydrogel and Its Application in Protected Quick Response Codes. Mater. Chem. Front. 2020, 4, 869–874. 10.1039/C9QM00699K.

[ref12] Gu L.; Shi H.; Bian L.; Gu M.; Ling K.; Wang X.; Ma H.; Cai S.; Ning W.; Fu L.; et al. Colour-Tunable Ultra-Long Organic Phosphorescence of a Single-Component Molecular Crystal. Nat. Photonics 2019, 13, 406–411. 10.1038/s41566-019-0408-4.

[ref13] Jeon S. O.; Lee K. H.; Kim J. S.; Ihn S.-G.; Chung Y. S.; Kim J. W.; Lee H.; Kim S.; Choi H.; Lee J. Y. High-Efficiency, Long-Lifetime Deep-Blue Organic Light-Emitting Diodes. Nat. Photonics 2021, 15, 208–215. 10.1038/s41566-021-00763-5.

[ref14] Zhang G.; Palmer G. M.; Dewhirst M. W.; Fraser C. L. A Dual-Emissive-Materials Design Concept Enables Tumour Hypoxia Imaging. Nat. Mater. 2009, 8, 747–751. 10.1038/nmat2509. 19668206 PMC2846459

[ref15] Jinnai K.; Kabe R.; Adachi C. Wide-Range Tuning and Enhancement of Organic Long-Persistent Luminescence Using Emitter Dopants. Adv. Mater. 2018, 30, 1800365 10.1002/adma.201800365. 30062742

[ref16] Wu S.; Min H.; Shi W.; Cheng P. Multicenter Metal-Organic Framework-Based Ratiometric Fluorescent Sensors. Adv. Mater. 2020, 32, 1805871 10.1002/adma.201805871. 30790371

[ref17] He Z.; Gao H.; Zhang S.; Zheng S.; Wang Y.; Zhao Z.; Ding D.; Yang B.; Zhang Y.; Yuan W. Z. Achieving Persistent, Efficient, and Robust Room-Temperature Phosphorescence from Pure Organics for Versatile Applications. Adv. Mater. 2019, 31, 1807222 10.1002/adma.201807222. 30907466

[ref18] Zhu X.; Xu Y.; Zhao C.; Jia C.; Guo X. Recent Advances in Photochemical Reactions on Single-Molecule Electrical Platforms. Macromol. Rapid Commun. 2022, 43, 2200017 10.1002/marc.202200017. 35150177

[ref19] Sun M. J.; Zhong Y. W.; Yao J. Thermal-Responsive Phosphorescent Nanoamplifiers Assembled from Two Metallophosphors. Angew. Chem., Int. Ed. 2018, 57, 7820–7825. 10.1002/anie.201803546. 29665184

[ref20] Wang J.-X.; Zhang H.; Niu L.-Y.; Zhu X.; Kang Y.-F.; Boulatov R.; Yang Q.-Z. Organic Composite Crystal with Persistent Room-Temperature Luminescence Above 650 nm by Combining Triplet-Triplet Energy Transfer with Thermally Activated Delayed Fluorescence. CCS. Chem. 2020, 2, 1391–1398. 10.31635/ccschem.020.202000158.

[ref21] Chen B.; Huang W.; Nie X.; Liao F.; Miao H.; Zhang X.; Zhang G. An Organic Host-Guest System Producing Room-Temperature Phosphorescence at the Parts-Per-Billion Level. Angew. Chem., Int. Ed. 2021, 60, 16970–16973. 10.1002/anie.202106204. 34080278

[ref22] Wang J.-X.; Yin J.; Shekhah O.; Bakr O. M.; Eddaoudi M.; Mohammed O. F. Energy Transfer in Metal-Organic Frameworks for Fluorescence Sensing. ACS Appl. Mater. Interfaces 2022, 10.1021/acsami.1c24759. PMC8895374 35175725

[ref23] Gan N.; Wang X.; Ma H.; Lv A.; Wang H.; Wang Q.; Gu M.; Cai S.; Zhang Y.; Fu L.; et al. Manipulating the Triplet Chromophore Stacking for Ultralong Organic Phosphorescence in Crystal. Angew. Chem., Int. Ed. 2019, 58, 14140–14145. 10.1002/anie.201907572. 31359548

[ref24] Li S.; Fu L.; Xiao X.; Geng H.; Liao Q.; Liao Y.; Fu H. Regulation of Thermally Activated Delayed Fluorescence to Room-Temperature Phosphorescent Emission Channels by Controlling the Excited-States Dynamics via J- and H-Aggregation. Angew. Chem., Int. Ed. 2021, 60, 18059–18064. 10.1002/anie.202103192. 34075684

[ref25] Liang S.; Wang Y.; Wu X.; Chen M.; Mu L.; She G.; Shi W. An Ultrasensitive Ratiometric Fluorescent Thermometer based on Frustrated Static Excimers in the Physiological Temperature Range. Chem. Commun. 2019, 55, 3509–3512. 10.1039/C9CC00614A. 30839036

[ref26] Wang J.-X.; Niu L.-Y.; Chen P.-Z.; Chen Y.-Z.; Yang Q.-Z.; Boulatov R. Ratiometric O_2_ Sensing based on Selective Self-Sensitized Photooxidation of Donor-Acceptor Fluorophores. Chem. Commun. 2019, 55, 7017–7020. 10.1039/C9CC03232K. 31150036

[ref27] Chen J.; Chen X.; Liu Y.; Li Y.; Zhao J.; Yang Z.; Zhang Y.; Chi Z. A Color-Tunable Single-Component Luminescent Molecule with Multiple Emission Centers. Chem. Sci. 2021, 12, 9201–9206. 10.1039/D1SC02094C. 34276951 PMC8261876

[ref28] Wang J.-X.; Fang Y.-G.; Li C.-X.; Niu L.-Y.; Fang W.-H.; Cui G.; Yang Q.-Z. Time-Dependent Afterglow Color in a Single-Component Organic Molecular Crystal. Angew. Chem., Int. Ed. 2020, 59, 10032–10036. 10.1002/anie.202001141. 32043718

[ref29] Shoji Y.; Ikabata Y.; Ryzhii I.; Ayub R.; El Bakouri O.; Sato T.; Wang Q.; Miura T.; Karunathilaka B. S. B.; Tsuchiya Y.; et al. An Element-Substituted Cyclobutadiene Exhibiting High-Energy Blue Phosphorescence. Angew. Chem., Int. Ed. 2021, 60, 21817–21823. 10.1002/anie.202106490. 34097333

[ref30] Wang X.; Hu J.; Lv J.; Yang Q.; Tian H.; Shao S.; Wang L.; Jing X.; Wang F. pi-Stacked Donor-Acceptor Dendrimers for Highly Efficient White Electroluminescence. Angew. Chem., Int. Ed. 2021, 60, 16585–16593. 10.1002/anie.202104145. 33942454

[ref31] Li Y.; Jiang L.; Liu W.; Xu S.; Li T. Y.; Fries F.; Zeika O.; Zou Y.; Ramanan C.; Lenk S.; et al. Reduced Intrinsic Non-Radiative Losses Allow Room-Temperature Triplet Emission from Purely Organic Emitters. Adv. Mater. 2021, 33, 2101844 10.1002/adma.202101844. PMC11469145 34365677

[ref32] Tian Y.; Yang J.; Liu Z.; Gao M.; Li X.; Che W.; Fang M.; Li Z. Multistage Stimulus-Responsive Room Temperature Phosphorescence Based on Host-Guest Doping Systems. Angew. Chem., Int. Ed. 2021, 60, 20259–20263. 10.1002/anie.202107639. 34236129

[ref33] Yan Z. A.; Lin X.; Sun S.; Ma X.; Tian H. Activating Room-Temperature Phosphorescence of Organic Luminophores via External Heavy-Atom Effect and Rigidity of Ionic Polymer Matrix. Angew. Chem., Int. Ed. 2021, 60, 19735–19739. 10.1002/anie.202108025. 34240799

[ref34] Liu K.; Huang K.; Lv A.; Ye W.; Yang Y.; Shen K.; Zhi J.; Wang H.; Zhang R.; Wang J.; et al. Tunable Microstructures of Ultralong Organic Phosphorescence Materials. Chem. Commun. 2021, 57, 7276–7279. 10.1039/D1CC01563J. 34196639

[ref35] Sifain A. E.; Lystrom L.; Messerly R. A.; Smith J. S.; Nebgen B.; Barros K.; Tretiak S.; Lubbers N.; Gifford B. J. Predicting Phosphorescence Energies and Inferring Wavefunction Localization with Machine Learning. Chem. Sci. 2021, 12, 10207–10217. 10.1039/D1SC02136B. 34447529 PMC8336587

[ref36] Takano S.; Hirai H.; Nakashima T.; Iwasa T.; Taketsugu T.; Tsukuda T. Photoluminescence of Doped Superatoms M@Au12 (M = Ru, Rh, Ir) Homoleptically Capped by (Ph_2_)PCH_2_P(Ph_2_): Efficient Room-Temperature Phosphorescence from Ru@Au12. J. Am. Chem. Soc. 2021, 143, 10560–10564. 10.1021/jacs.1c05019. 34232036

[ref37] Wang Y.; Peng Q.; Shuai Z. A Computational Scheme for Evaluating the Phosphorescence Quantum Efficiency: Applied to Blue-Emitting Tetradentate Pt(ii) Complexes. Mater. Horiz. 2022, 9, 334–341. 10.1039/D1MH00552A. 34842258

[ref38] Ma X.; Xu C.; Wang J.; Tian H. Amorphous Pure Organic Polymers for Heavy-Atom-Free Efficient Room-Temperature Phosphorescence Emission. Angew. Chem., Int. Ed. 2018, 57, 10854–10858. 10.1002/anie.201803947. 29719096

[ref39] Zhao W.; Cheung T. S.; Jiang N.; Huang W.; Lam J. W. Y.; Zhang X.; He Z.; Tang B. Z. Boosting the Efficiency of Organic Persistent Room-Temperature Phosphorescence by Intramolecular Triplet-Triplet Energy Transfer. Nat. Commun. 2019, 10, 1595 10.1038/s41467-019-09561-8. 30962451 PMC6453937

[ref40] Picarra S.; Duhamel J.; Fedorov A.; Martinho J. M. G. Coil–Globule Transition of Pyrene-Labeled Polystyrene in Cyclohexane: Determination of Polymer Chain Radii by Fluorescence. J. Phys. Chem. B 2004, 108, 12009 10.1021/jp048616o.

[ref41] Dimitriev O. P.; Piryatinski Y. P.; Slominskii Y. L. Excimer Emission in J-Aggregates. J. Phys. Chem. Lett. 2018, 9, 2138–2143. 10.1021/acs.jpclett.8b00481. 29634281

[ref42] Wang Y.; Chen J.; Chen Y.; Li W.; Yu C. Polymer-Induced Perylene Probe Excimer Formation and Selective Sensing of DNA Methyltransferase Activity Through the Monomer-Excimer Transition. Anal. Chem. 2014, 86, 4371–4378. 10.1021/ac500195u. 24697780

[ref43] Lohar S.; Safin D. A.; Sengupta A.; Chattopadhyay A.; Matalobos J. S.; Babashkina M. G.; Robeyns K.; Mitoraj M. P.; Kubisiak P.; Garcia Y.; et al. Ratiometric Sensing of Lysine Tthrough the Formation of the Pyrene Excimer: Experimental and Computational Studies. Chem. Commun. 2015, 51, 8536–8539. 10.1039/C5CC01359C. 25893984

[ref44] Chen P.-Z.; Niu L.-Y.; Zhang H.; Chen Y.-Z.; Yang Q.-Z. Exploration of the Two-Step Crystallization of Organic Micro/Nano Crystalline Materials by Fluorescence Spectroscopy. Mater. Chem. Front. 2018, 2, 1323–1327. 10.1039/C8QM00118A.

[ref45] Chen P.-Z.; Zhang H.; Niu L.-Y.; Zhang Y.; Chen Y.-Z.; Fu H.-B.; Yang Q.-Z. A Solid-State Fluorescent Material Based on Carbazole-Containing Difluoroboron β-Diketonate: Multiple Chromisms, the Self-Assembly Behavior, and Optical Waveguides. Adv. Funct. Mater. 2017, 27, 1700332 10.1002/adfm.201700332.

[ref46] Liu N.; Chen P.-Z.; Wang J.-X.; Niu L.-Y.; Yang Q.-Z. Difluoroboron β-Diketonate Dye with Intense Red/Near-Infrared Fluorescence in Solutions and Solid States. Chin. Chem. Lett. 2019, 30, 1939–1941. 10.1016/j.cclet.2019.04.058.

[ref47] Wang J.-X.; Yu Y.-S.; Niu L.-Y.; Zou B.; Wang K.; Yang Q.-Z. A Difluoroboron Beta-Diketonate based Thermometer with Temperature-Dependent Emission Wavelength. Chem. Commun. 2020, 56, 6269–6272. 10.1039/D0CC01505A. 32373809

[ref48] Zhu J.-Y.; Li C.-X.; Chen P.-Z.; Ma Z.; Zou B.; Niu L.-Y.; Cui G.; Yang Q.-Z. A Polymorphic Fluorescent Material with Strong Solid State Emission and Multi-Stimuli-Responsive Properties. Mater. Chem. Front. 2020, 4, 176–181. 10.1039/C9QM00518H.

[ref49] Paisley N. R.; Halldorson S. V.; Tran M. V.; Gupta R.; Kamal S.; Algar W. R.; Hudson Z. M. Near-Infrared-Emitting Boron-Difluoride-Curcuminoid-Based Polymers Exhibiting Thermally Activated Delayed Fluorescence as Biological Imaging Probes. Angew. Chem., Int. Ed. 2021, 60, 18630–18638. 10.1002/anie.202103965. 34133838

[ref50] Wang J.-X.; Zhang T.-S.; Zhu X.; Li C.-X.; Dong L.; Cui G.; Yang Q.-Z. Organic Thermometers Based on Aggregation of Difluoroboron beta-Diketonate Chromophores. J. Phys. Chem. A 2020, 124, 10082–10089. 10.1021/acs.jpca.0c08649. 33226240

[ref51] Xu W.; Yu Y.; Ji X.; Zhao H.; Chen J.; Fu Y.; Cao H.; He Q.; Cheng J. Self-Stabilized Amorphous Organic Materials with Room-Temperature Phosphorescence. Angew. Chem., Int. Ed. 2019, 58, 16018–16022. 10.1002/anie.201906881. 31419005

[ref52] Sun S.; Wang J.; Ma L.; Ma X.; Tian H. A Universal Strategy for Organic Fluid Phosphorescence Materials. Angew. Chem., Int. Ed. 2021, 60, 18557–18560. 10.1002/anie.202107323. 34133818

[ref53] Yang J.; Zhen X.; Wang B.; Gao X.; Ren Z.; Wang J.; Xie Y.; Li J.; Peng Q.; Pu K.; et al. The Influence of the Molecular Packing on the Room Temperature Phosphorescence of Purely Organic Luminogens. Nat. Commun. 2018, 9, 840 10.1038/s41467-018-03236-6. 29483501 PMC5826932

[ref54] Becke A. D. Density-Functional Exchange-Energy Approximation with Correct Asymptotic Behavior. Phys. Rev. A 1988, 38, 3098–3100. 10.1103/PhysRevA.38.3098. 9900728

[ref55] Lee C.; Yang W.; Parr R. G. Development of the Colle-Salvetti Correlation-Energy Formula into a Functional of the Electron Density. Phys. Rev. B 1988, 37, 785 10.1103/PhysRevB.37.785. 9944570

[ref56] Vosko S. H.; Wilk L.; Nusair M. Accurate Spin-Dependent Electron Liquid Correlation Energies for Local Spin Density Calculations: A Critical Analysis. J. Phys. (Paris) 1980, 58, 1200–1211. 10.1139/p80-159.

[ref57] Becke A. D. A New Mixing of Hatree-Fock and Local Densityfunctional Theories. J. Chem. Phys. 1993, 98, 1372–1377. 10.1063/1.464304.

[ref58] Marques M.; Rubio A.; Gross E. K.; Burke K.; Nogueira F.; Ullrich C. A. Time-Dependent Density Functional Theory; Springer Science & Business Media, 2006.

[ref59] Barone V.; Cossi M. Quantum Calculation of Molecular Energies and Energy Gradients in Solution by a Conductor Solvent Model. J. Phys. Chem. A 1998, 102, 1995–2001. 10.1021/jp9716997.

[ref60] Andersson K.; Malmqvist P.-Å.; Roos B. O.; Sadlej A. J.; Wolinski K. Second-Order Perturbation Theory with a CASSCF Reference Function. J. Phys. Chem. A 1990, 94, 5483–5488. 10.1021/j100377a012.

[ref61] Andersson K.; Malmqvist P.-Å.; Roos B. O. Second-Order Perturbation Theory with a Complete Active Space Self-Consistent Field Reference Function. J. Chem. Phys. 1992, 96, 1218–1226. 10.1063/1.462209.

[ref62] Maseras F.; Morokuma K. IMOMM: A New Integrated Ab Initio + Molecular Mechanics Geometry Optimization Scheme of Equilibrium Structures and Transition States. J. Comput. Chem. 1995, 16, 1170 10.1002/jcc.540160911.

[ref63] Vreven T.; Morokuma K.; Farkas O.; Schlegel H. B.; Frisch M. J. Geometry Optimization with QM/MM, ONIOM, and Other Combined Methods. I. Microiterations and Constraints. J. Comput. Chem. 2003, 24, 760 10.1002/jcc.10156. 12666168

