# Supplementary material for: Tunable Fluorescence and Afterglow in Organic Crystals for Temperature Sensing
Source: J Phys Chem Lett. 2022 Feb 21;13(8):1985–90. doi: 10.1021/acs.jpclett.2c00168 (PMC8900125; doi:10.1021/acs.jpclett.2c00168)
Supplement: Supplementary file 1 — jz2c00168_si_001.pdf [file jz2c00168_si_001.pdf]

Supporting Information

Tunable Fluorescence and Afterglow in Organic Crystals for Temperature Sensing

Jian-Xin Wang,[1,3*#] Ling-Ya Peng,[2#] Zheng-Fei Liu,[1] Xin Zhu,[1] Li-Ya Niu,[1] Ganglong Cui,[2] Qing-Zheng Yang[1]

[1]Key Laboratory of Radiopharmaceuticals, Ministry of Education, College of Chemistry, Beijing Normal University, Beijing 100875, P. R. China.

[2]Key Laboratory of Theoretical and Computational Photochemistry, Ministry of Education, College of Chemistry, Beijing Normal University, Beijing 100875, P. R. China.

[3]Advanced Membranes and Porous Materials Center, Division of Physical Science and Engineering, King Abdullah University of Science and Technology, Thuwal 23955-6900, Kingdom of Saudi Arabia

Materials and Methods

All chemicals were purchased from commercial suppliers and used without further purification. NMR spectra were recorded on JEOL-400 or JEOL-600 spectrometers. High-resolution mass spectra were measured on a Bruker Solarix XR Fourier Transform Ion Cyclotron Resonance Mass Spectrometer. Absorption and photoluminescence spectra for solutions were recorded on Hitachi UV-3900 and F-4600 spectrometer. The single-crystal structures were measured on the XtaLAB PRO 007HF (Mo) single crystal X-ray diffractometer. The temperature-dependent fluorescence and phosphorescence spectra were performed on Edinburgh FLS980 Spectrometer with Oxford variable temperature equipment. The temperature could be turned from 300 K to 77 K. After reaching the set temperature and the temperature was stable for a few minutes, the corresponding spectral tests were carried out.

Synthesis and characterization

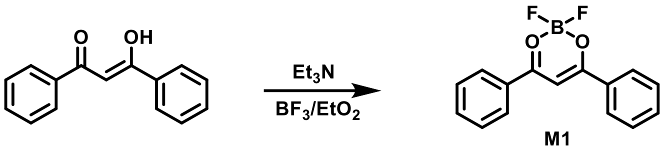

Synthesis of M1.[1,2] To a solution of the β-diketonates (1 mmol) in 30 mL CH_2Cl_2 was added Et_3N (5 mmol) and BF_3/Et_2O (10 mmol) at room temperature. After stirring 2 h at room temperature in the dark, 100 mL of water was added. The organic layers were collected, washed with saturated aqueous NH_4Cl, and dried over anhydrous Na_2SO_4, followed by filtration and evaporation of the solvent. Column chromatography using CH_2Cl_2/petroleum ether (1:1, v/v) as the eluent to afford the products (yield: 92 %). ^{1}H NMR (600 MHz, *Chloroform-d*) δ 8.15 (d, *J* = 8.2 Hz, 4 H), 7.74 – 7.65 (m, 2 H), 7.56 (t, *J* = 7.9 Hz, 4 H), 7.19 (S, 1 H). ^{13}C NMR (150 MHz, *Chloroform-d*) δ 183.4, 135.4, 132.1, 129.3, 129.0, 93.5. HRMS: calc. for [M+Na$^+$] 295.0712, found: 295.0712.

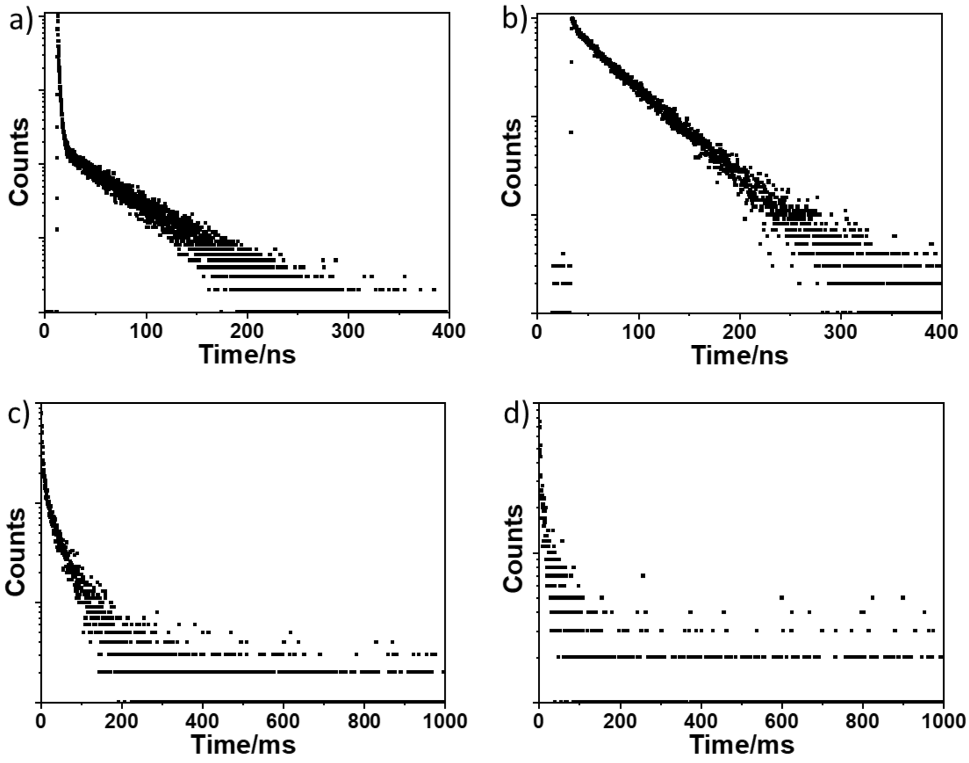

Figure S1. a) -d) Time-resolved decay profiles of M1 crystals monitored at 470 nm (40.8 ns), 540 nm (85.1 ns), 560 nm (40.6 ms) and 650 nm (15.3 ms), respectively.

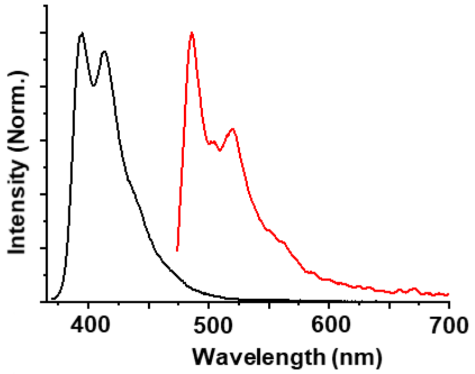

Figure S2. Prompt (black line, at room temperature) and delayed (red line, delayed 1 ms, at 77 K) emission spectra of M1 in 1 uM chloroform solution.

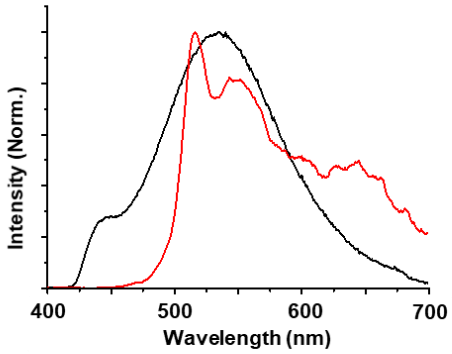

Figure S3. Prompt (black line, at room temperature) and delayed (red line, delayed 1 ms, at 77 K) emission spectra of M1 in 200 mM chloroform solutions.

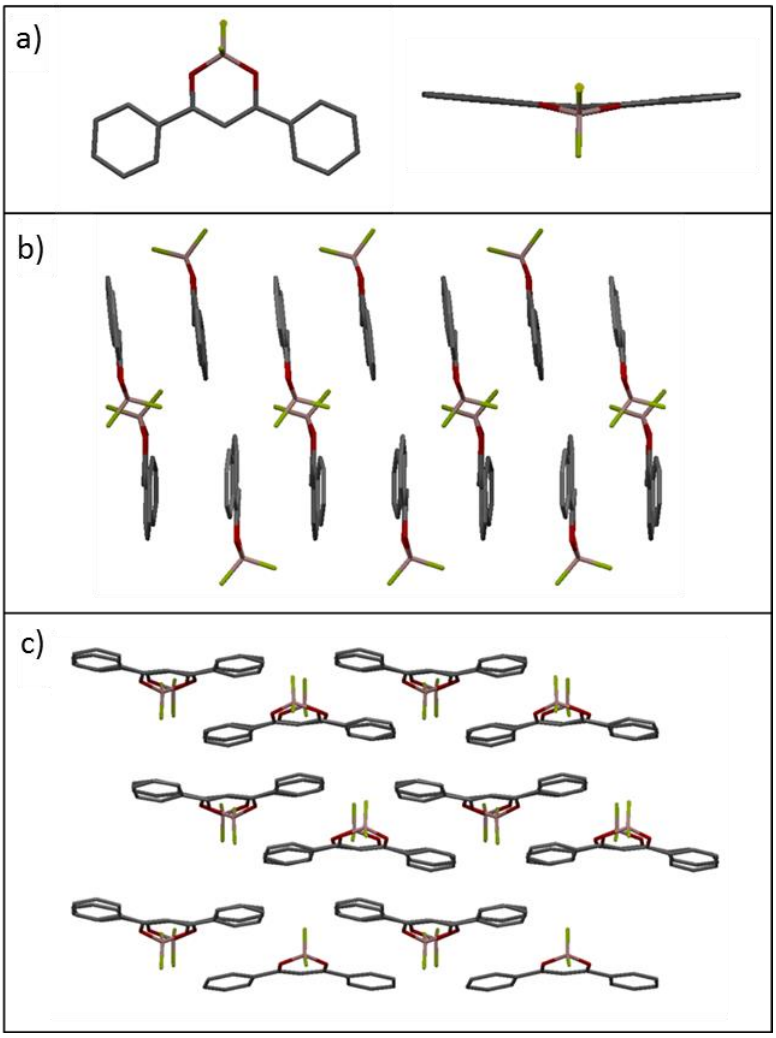

Figure S4. a) The molecular conformation of M1 in front view and side view, respectively. b)-c) Molecular stacking structures along the short and long molecule axis, respectively (hydrogen atoms are removed for clarity).

Table S1. The summarized crystal data of M1 from 300 K to 100 K.

Empirical formula	$C_{15}H_{11}BF_2O_2$	$C_{15}H_{11}BF_2O_2$	$C_{15}H_{11}BF_2O_2$
CCDC number	1977300	1977289	1977283
Temperature/K	300	200	100
Formula weight	272.05	272.05	272.05
Crystal system	monoclinic	monoclinic	monoclinic
Space group	$P2_1/n$	$P2_1/n$	$P2_1/n$
Unit cell dimensions	a = 13.7959 (9) Å<br>b = 14.0020 (8) Å<br>c = 14.1100 (11) Å<br>α= 90°<br>β= 107.154 (3)°<br>γ= 90°	a = 13.7674 (7) Å<br>b = 13.9418 (6) Å<br>c = 13.8838 (8) Å<br>α= 90°<br>β= 107.122 (6)°<br>γ= 90°	a = 13.7348 (5) Å<br>b = 13.8846 (5) Å<br>c = 13.6954 (6) Å<br>α= 90°<br>β= 107.170(4)°<br>γ= 90°
Volume (Å^3)	2604.4 (3)	2546.8 (2)	2495.3 (18)
Z	8	8	8
ρ_{calc} g/cm^3	1.388	1.419	1.448

HRMS

Peking University Mass Spectrometry Sample Analysis Report

Analysis Info

Analysis Name	FTMS-19120378_Pos_20191230_000003.d	Acquisition Date	12/30/2019 4:40:43 PM
Sample	2	Instrument	Bruker Solarix XR FTMS
Comment		Operator	Peking University

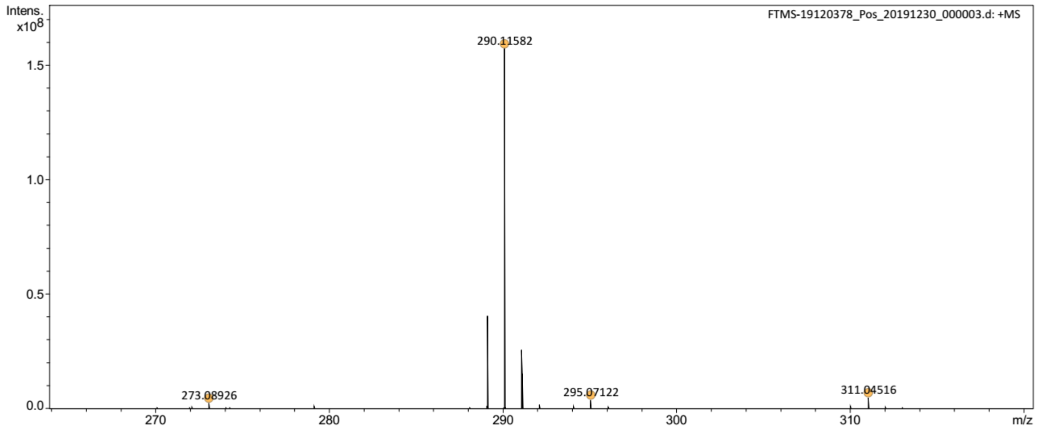

Meas. m/z	#	Ion Formula	Score	m/z	err [ppm]	Mean err [ppm]	mSigma	rdb	e$^-$ Conf	N-Rule
273.089258	1	C15H12BF2O2	100.00	273.089293	0.1	0.1	15.8	10.0	even	ok
290.115817	1	C15H15BF2NO2	100.00	290.115842	0.1	0.1	17.0	9.0	even	ok
295.071225	1	C15H11BF2NaO2	100.00	295.071237	0.0	0.0	22.7	10.0	even	ok
311.045157	1	C15H11BF2KO2	100.00	311.045174	0.1	-1.5	18.1	10.0	even	ok

Figure S5. High-resolution mass spectrum of M1**.**

NMR spectra

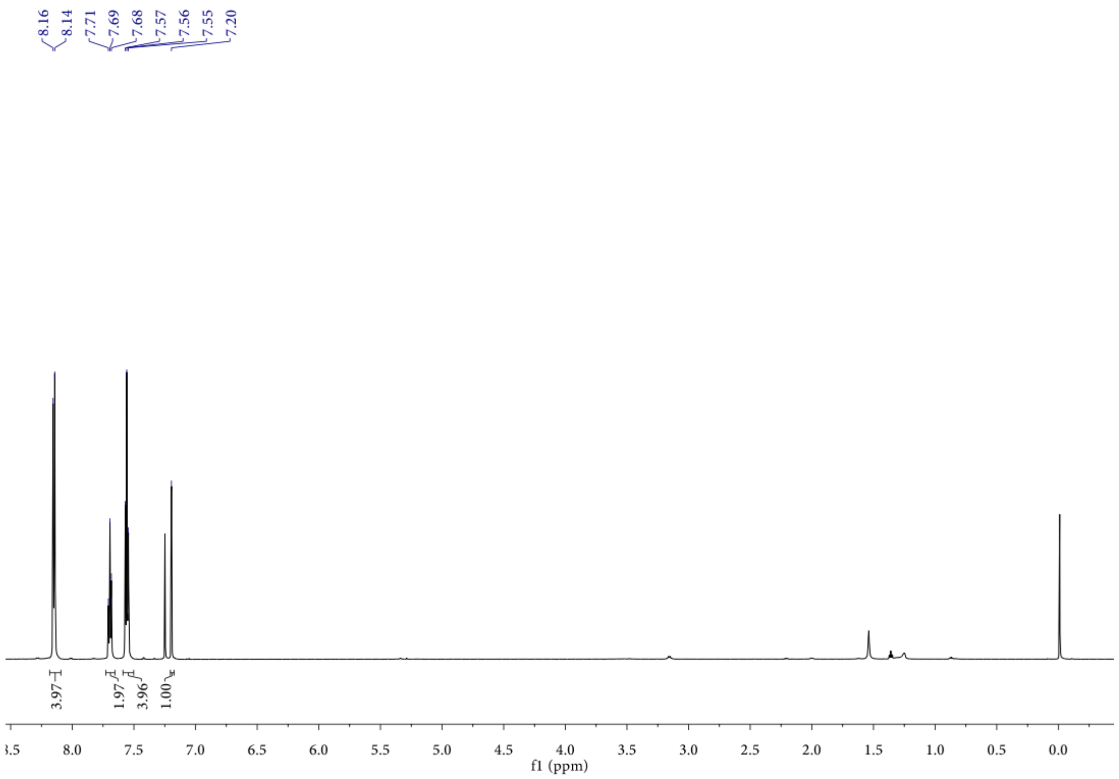

Figure S6. ^{1}H NMR spectrum of M1 in $CDCl_3$ (500μL).

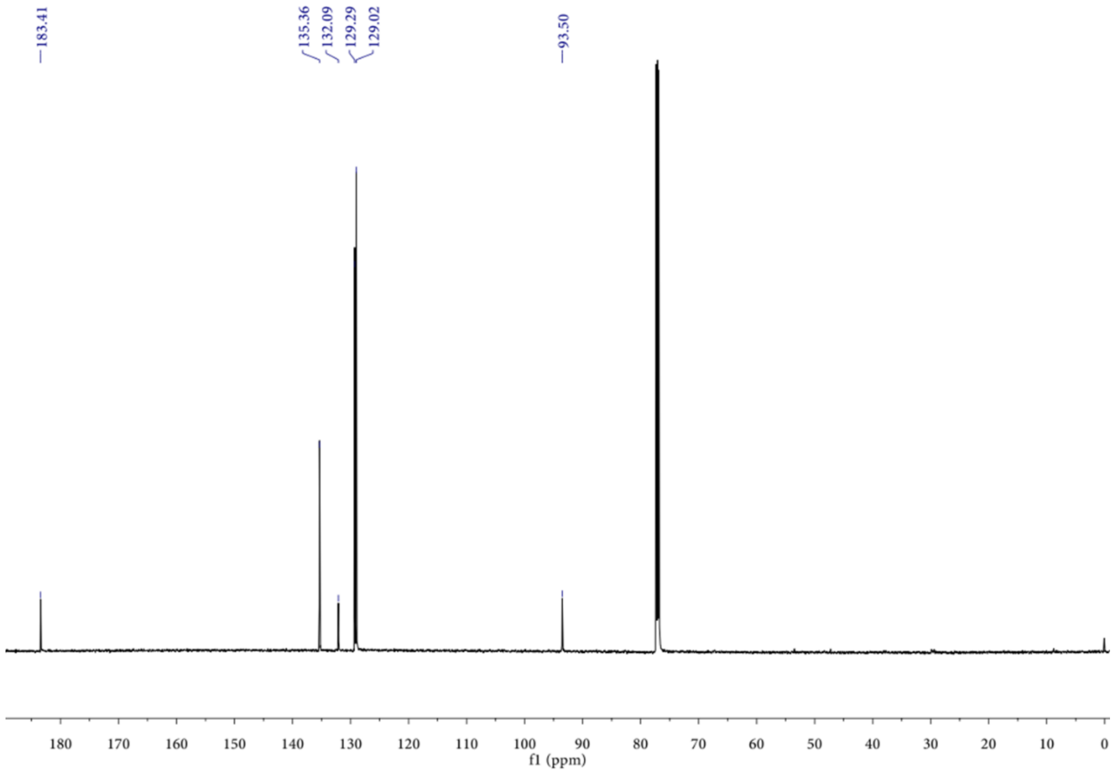

Figure S7. ^{13}C NMR spectrum of M1 in $CDCl_3$ (500μL).

Computational Details

Geometry optimizations are performed at the B3LYP level for the S_0 structures and at the TD-B3LYP level for the S_1 and T_1 structures in gas phase and solution.[3-5] In all the DFT and TD-DFT calculations, the 6-31G* basis sets are used.[6-7] A polarizable continuum medium (PCM) model with default parameters is used to consider solvation effects of chloroform.[8,9] For the corresponding situation in the crystal, the ONIOM method is adopted.[10,11] For the monomer case, the innermost molecule is treated as the QM region and described using the B3LYP (S_0) and TD-B3LYP (S_1 and T_1) methods; while the outermost molecules are treated using the efficient universal force field (UFF) method.[12] For the dimer case, the central two molecules are chosen as the QM region and treated with the same methods as those for the above monomer case; whereas, the surrounding molecules are chosen as the MM region. The MM charges that are calculated using the QEQ formalism[13] are embedded into the QM electronic structure calculations to consider the polarization interaction of the MM charges on the QM atoms. All the geometry optimizations in gas phase and solution are carried out using Gaussian09 package;[14] all the ONIOM geometry optimizations are carried out using Gaussian16 package.[15]

The energies of all the optimized structures are further refined at the CASPT2, CASPT2/PCM, and QM(CASPT2)/MM levels.[16,17] In all CASPT2 calculations, an active space of ten electrons in nine molecular orbitals is used for the monomer case, an active space of twelve electrons in ten molecular orbitals is chosen for the dimer case. The ANO-RCC-VDZP basis sets are selected.[18] Moreover, the Cholesky decomposition technique with unbiased auxiliary basis sets is adopted to accelerate computations;[19] the imaginary shift technique (0.2 au) is employed to avoid the intruder-state issue;[20] the ionization potential electron affinity (IPEA) shift is set to zero.[21] The same computational parameters are used for the situation in chloroform solution in which the polarizable continuum medium (PCM) model is used to consider solvent effects.[22,23] In the QM(CASPT2)/MM calculations for the crystal situation, the MM charges presented in the Hamiltonian of the QM region are extracted from the ONIOM calculations. All the CASPT2, CASPT2/PCM, and QM(CASPT2)/MM calculations are performed by MOLCAS8.0.[24,25]

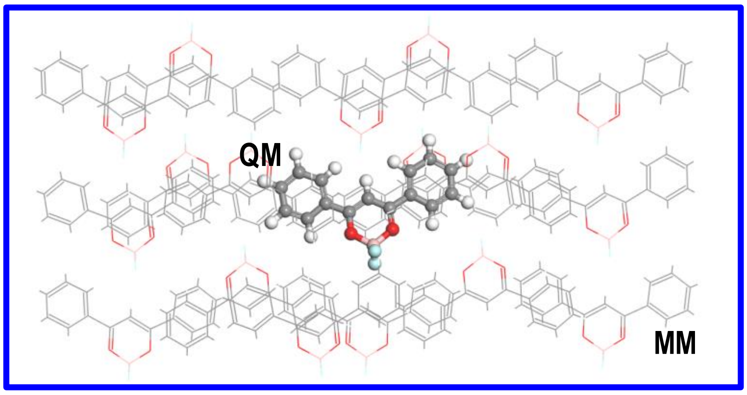

Figure S8. ONIOM model of **M1** in monomer in which the inner molecule is treated quantum mechanically and the outermost molecules are described molecule mechanically. The electronic embedding scheme is used to consider the polarization effects of the MM charges on the QM region.

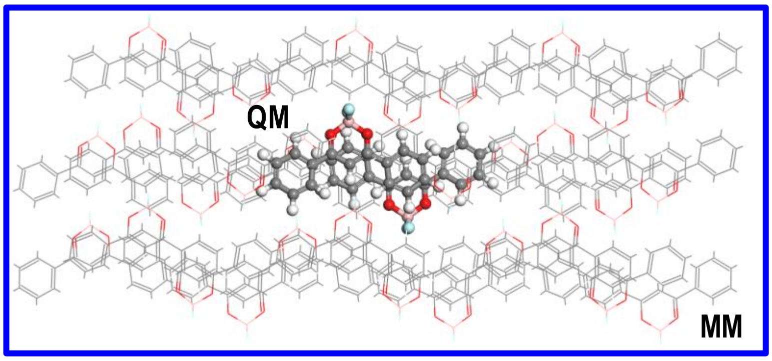

Figure S9. ONIOM model of **M1** in dimer. The central chromophore is chosen as the QM region and the left ones are treated as the MM region frozen in geometry optimizations.

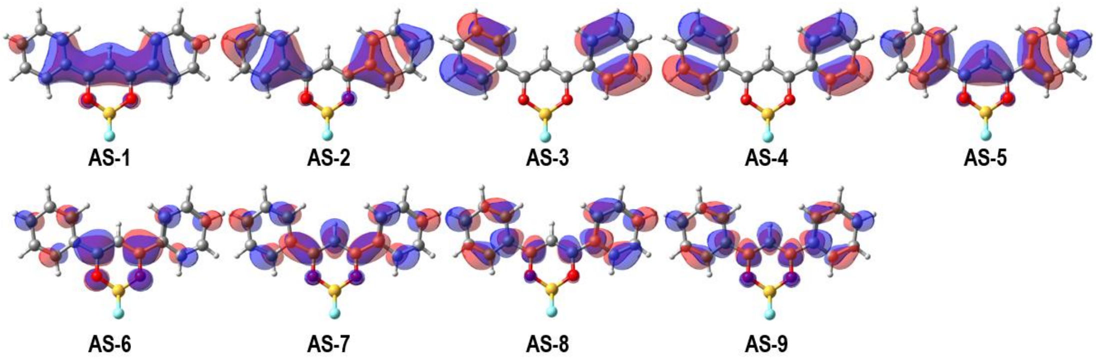

Figure S10. Molecular orbitals used as active space of **M1** in monomer in CASPT2/PCM calculations.

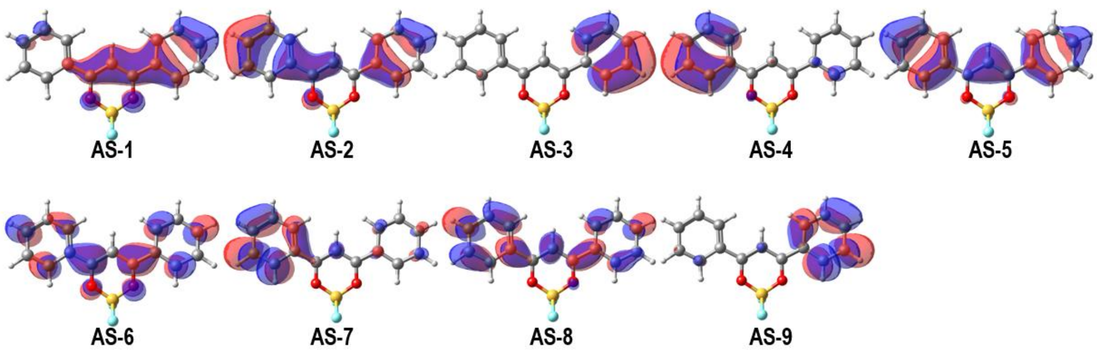

Figure S11. Molecular orbitals used as active space of **M1** in monomer in QM(CASPT2)/MM calculations.

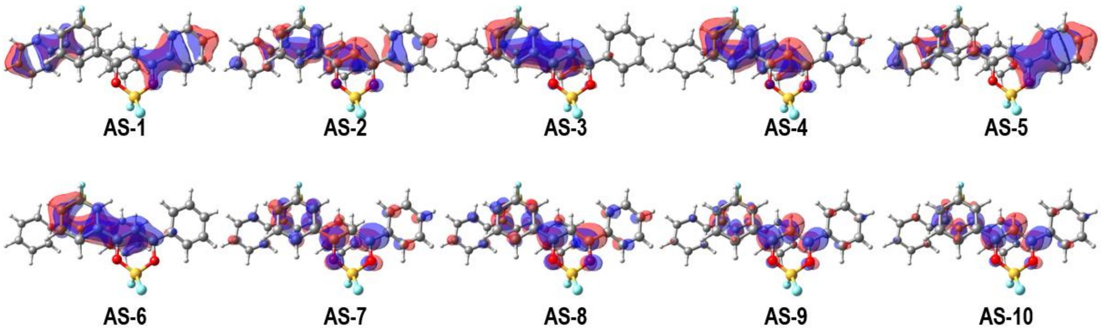

Figure S12. Molecular orbitals used as active space of **M1** in dimer in QM(CASPT2)/MM calculations.

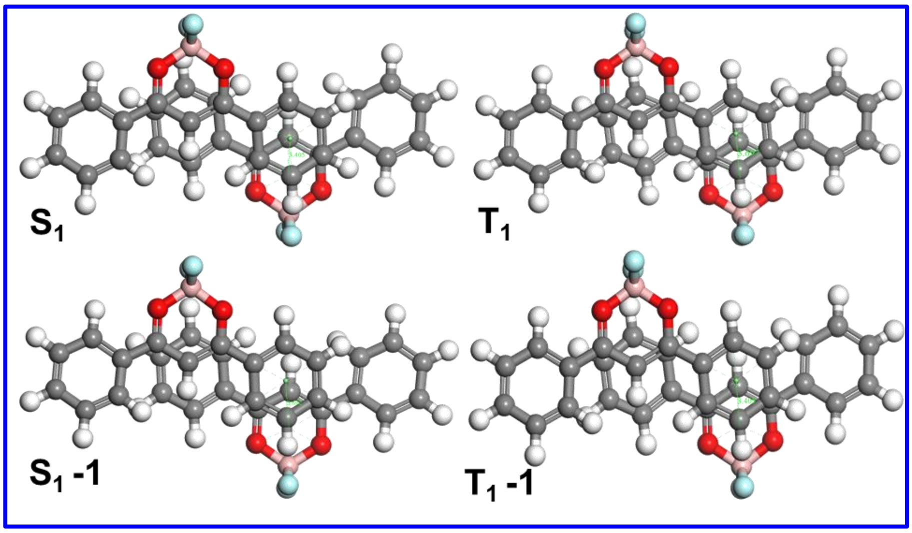

Figure S13. S_1 and T_1 in dimer in the solid phase (QM(CASPT2)/MM), S_1-1 and T_1-1 (S_1 and T_1 in dimer when changed the centroid distance of two aromatic nucleuses).

Table S2. Vertical excitation energy (kcal mol^{-1}) involved $S_0 \rightarrow S_1$ electronic transition computed at 5-Root state averaged CASPT2/PCM level of monomer.

	Monomer
CASPT2/PCM	76.9 (372 nm)

Table S3. Adiabatic excitation energy (kcal mol^{-1}) involved $S_0 \rightarrow S_1$ electronic transition computed at 5-Root state averaged CASPT2/PCM level of monomer.

	Monomer
CASPT2/PCM	75.4 (379 nm)

Table S4. Vertical emission energies (kcal mol^{-1}) involved $S_1 \rightarrow S_0$ and $T_1 \rightarrow S_0$ electronic transition computed at 5-Root state averaged CASPT2/PCM level of monomer.

	Monomer
$S_1 \rightarrow S_0$	70.0 (408 nm)
$T_1 \rightarrow S_0$	52.6 (543 nm)

Table S5. Vertical excitation energies (kcal mol^{-1}) involved $S_0 \rightarrow S_1$ electronic transition computed at 5-Root state averaged QM(CASPT2)/MM level.

	QM(CASPT2)/MM
Monomer	70.3 (407 nm)
Dimer	63.6 (449 nm)

Table S6. Adiabatic excitation energies (kcal mol^{-1}) involved $S_0 \rightarrow S_1$ electronic transition computed at 5-Root state averaged QM(CASPT2)/MM level.

	QM(CASPT2)/MM
Monomer	66.5 (430 nm)
Dimer	59.6 (480 nm)

Table S7. Vertical emission energies (kcal mol^{-1}) involved $S_1 \rightarrow S_0$ electronic transition computed at 5-Root state averaged QM(CASPT2)/MM level.

	QM(CASPT2)/MM
Monomer	64.6 (443 nm)
Dimer	53.3 (536 nm)

Table S8. Vertical emission energies (kcal mol^{-1}) involved $T_1 \rightarrow S_0$ electronic transition computed at 5-Root state averaged QM(CASPT2)/MM level.

	QM(CASPT2)/MM
Monomer	52.3 (547 nm)
Dimer	52.1 (548 nm)

Table S9. Vertical emission energies (kcal mol^{-1}) involved $S_1 \rightarrow S_0$ and $T_1 \rightarrow S_0$ electronic transition computed at 5-Root state averaged QM(CASPT2)/MM level of dimer when changed the centroid distance of two aromatic nucleuses.

Distance (Å)	$\mathbf{S_1 \rightarrow S_0}$	$\mathbf{T_1 \rightarrow S_0}$
3.80	-	52.2 (548 nm)
3.70	53.3 (536 nm)	-
3.40	51.8 (552 nm)	45.6 (627 nm)

Cartesian coordinates of the optimized structures in gas state (unit: angstrom).

monomer.S_0

O	-1.22797380	1.48298654	-0.26544881
O	1.22794700	1.48284820	-0.26563306
F	0.00017214	2.81584138	1.21645713
F	0.00004535	3.31695916	-1.03232497
C	-1.20658709	0.20542473	-0.04229723
C	2.52449915	-0.45765612	-0.01146534
C	-2.52455116	-0.45754278	-0.01154792
C	1.20654858	0.20532344	-0.04230590
C	-2.65939750	-1.85043272	-0.14334180
H	-1.78485008	-2.47217626	-0.30507953
C	-0.00004871	-0.47829997	0.14330214
H	-0.00015869	-1.52527231	0.40230228
C	-3.67774883	0.33018029	0.14985505
H	-3.56947223	1.40464694	0.24575871
C	2.65926645	-1.85072164	-0.14145801
H	1.78464658	-2.47272096	-0.30177775
C	3.67780748	0.33024543	0.14824677
H	3.56961920	1.40483630	0.24277044
C	-4.93459461	-0.26612910	0.19484944
H	-5.81883049	0.35045107	0.32786771
C	-3.91869185	-2.44211257	-0.10323178
H	-4.01252648	-3.51857994	-0.21386490
C	-5.05844784	-1.65219262	0.06991306
H	-6.04043034	-2.11610743	0.10281621
C	5.05842928	-1.65224462	0.07018834
H	6.04042214	-2.11613472	0.10317585
C	3.91855788	-2.44237316	-0.10121145
H	4.01230134	-3.51900075	-0.21035096
C	4.93466742	-0.26601329	0.19329216
H	5.81899184	0.35073726	0.32492546
B	0.00005846	2.35494128	-0.07276067

monomer.S_1

O	-1.231820737	1.523731196	-0.224014100
O	1.231823949	1.523733323	-0.224004801
F	-0.000005902	2.666622860	1.407160158
F	0.000001899	3.422982522	-0.774867980
C	-1.245312339	0.226749840	-0.045839387
C	2.522097923	-0.441265554	-0.034677112
C	-2.522096938	-0.441264647	-0.034673620
C	1.245310349	0.226749882	-0.045840333
C	-2.649355097	-1.858980768	0.057722171
H	-1.767620819	-2.488098972	0.120175836

C	0.000002081	-0.462965049	0.112081265
H	0.000003029	-1.513822749	0.352859067
C	-3.711351695	0.334423208	-0.131545966
H	-3.621731500	1.412228649	-0.201821290
C	2.649354547	-1.858983033	0.057685035
H	1.767618771	-2.488102322	0.120107241
C	3.711352065	0.334425013	-0.131517729
H	3.621732157	1.412232052	-0.201768499
C	-4.952333829	-0.277552286	-0.128673512
H	-5.850721780	0.329396451	-0.198882946
C	-3.899586641	-2.457354229	0.057394300
H	-3.976939967	-3.538803245	0.127610797
C	-5.059896889	-1.676766753	-0.033661908
H	-6.037559503	-2.150096313	-0.030816984
C	5.059896973	-1.676766478	-0.033664959
H	6.037559767	-2.150095752	-0.030819288
C	3.899586091	-2.457356833	0.057357780
H	3.976938787	-3.538807584	0.127547340
C	4.952334935	-0.277550116	-0.128644859
H	5.850723526	0.329400553	-0.198829061
B	-0.000000262	2.357030293	0.069430482

monomer.T_1

O	-1.235344798	1.529892761	-0.241736690
O	1.235342925	1.529895132	-0.241736523
F	-0.000002145	2.638341950	1.409397138
F	-0.000005219	3.435433099	-0.759409162
C	-1.254199384	0.225613935	-0.078923786
C	2.529066383	-0.446459168	-0.047121776
C	-2.529062594	-0.446462925	-0.047117592
C	1.254201236	0.225617759	-0.078928127
C	-2.659314446	-1.859154809	0.031416294
H	-1.781203926	-2.495776175	0.065309514
C	0.000002920	-0.459937231	0.042959530
H	0.000005312	-1.524775130	0.223662793
C	-3.715512330	0.332087124	-0.105944516
H	-3.624579278	1.410358717	-0.165621143
C	2.659314832	-1.859151142	0.031394017
H	1.781202164	-2.495771000	0.065268096
C	3.715516611	0.332090231	-0.105929785
H	3.624585692	1.410362696	-0.165592886
C	-4.960307090	-0.275024092	-0.079850200
H	-5.856422025	0.337777992	-0.121293923
C	-3.912217081	-2.454761303	0.057568353
H	-3.989759735	-3.536627898	0.118376313
C	-5.071362816	-1.671416020	0.003003797

H	-6.049862133	-2.142100593	0.024375417
C	5.071363673	-1.671415834	0.003003729
H	6.049862133	-2.142102133	0.024377610
C	3.912216535	-2.454760160	0.057548283
H	3.989756904	-3.536627723	0.118341722
C	4.960310826	-0.275023070	-0.079833361
H	5.856426840	0.337778416	-0.121262072
B	-0.000006879	2.356832894	0.066496664

Cartesian coordinates of the optimized structures in solution (unit: angstrom).

monomer.S_0

O	-1.225459000	1.487414000	-0.245494000
O	1.225460000	1.487415000	-0.245496000
F	0.000001000	2.858707000	1.204854000
F	-0.000002000	3.302421000	-1.049323000
C	-1.207288000	0.201000000	-0.034630000
C	2.523283000	-0.457925000	-0.007744000
C	-2.523283000	-0.457925000	-0.007739000
C	1.207288000	0.201001000	-0.034635000
C	-2.654764000	-1.853200000	-0.132867000
H	-1.780087000	-2.476913000	-0.283103000
C	0.000001000	-0.481499000	0.141234000
H	0.000003000	-1.532132000	0.383193000
C	-3.679711000	0.329403000	0.142848000
H	-3.578212000	1.404502000	0.237676000
C	2.654766000	-1.853196000	-0.132907000
H	1.780091000	-2.476904000	-0.283173000
C	3.679708000	0.329401000	0.142880000
H	3.578206000	1.404498000	0.237734000
C	-4.936045000	-0.268192000	0.181964000
H	-5.821886000	0.347356000	0.306703000
C	-3.913907000	-2.445410000	-0.098443000
H	-4.005269000	-3.522322000	-0.203288000
C	-5.056376000	-1.655707000	0.062380000
H	-6.037671000	-2.120735000	0.090773000
C	5.056375000	-1.655706000	0.062376000
H	6.037670000	-2.120735000	0.090769000
C	3.913909000	-2.445406000	-0.098483000
H	4.005274000	-3.522314000	-0.203357000
C	4.936042000	-0.268194000	0.181997000
H	5.821881000	0.347350000	0.306765000
B	0.000000000	2.341779000	-0.072391000

monomer.S_1

O	-1.227439430	1.526614820	-0.236044195

O	1.227439372	1.526614698	-0.236044514
F	0.000000242	2.640584709	1.423311012
F	0.000000000	3.439000363	-0.735921805
C	-1.239404445	0.216467370	-0.066497587
C	2.518627335	-0.443394006	-0.045613245
C	-2.518627393	-0.443393967	-0.045613701
C	1.239404387	0.216467312	-0.066497334
C	-2.654005861	-1.862469052	0.057610504
H	-1.776661691	-2.496087030	0.121646197
C	-0.000000058	-0.474646721	0.065417639
H	-0.000000149	-1.536116531	0.252374522
C	-3.707010234	0.338567681	-0.138920344
H	-3.614979305	1.415300370	-0.216494232
C	2.654005914	-1.862468983	0.057612431
H	1.776661823	-2.496086919	0.121649500
C	3.707010107	0.338567563	-0.138921463
H	3.614979103	1.415300158	-0.216496539
C	-4.951167617	-0.265687373	-0.123263192
H	-5.847073262	0.344561176	-0.191336219
C	-3.906565706	-2.452471717	0.071947014
H	-3.990245726	-3.532315870	0.151644702
C	-5.064528835	-1.664629353	-0.017290807
H	-6.044520194	-2.132480346	-0.004526189
C	5.064528830	-1.664629327	-0.017290891
H	6.044520194	-2.132480298	-0.004526569
C	3.906565780	-2.452471611	0.071948641
H	3.990245875	-3.532315680	0.151647409
C	4.951167511	-0.265687452	-0.123264567
H	5.847073103	0.344561039	-0.191338921
B	0.000000049	2.329467201	0.073556913

monomer.T_1

O	-1.231656830	1.532775364	-0.226685400
O	1.231656787	1.532775433	-0.226685545
F	0.000000041	2.672061797	1.409663849
F	-0.000000086	3.429884528	-0.765119985
C	-1.251374059	0.219847005	-0.072717055
C	2.525619206	-0.447098361	-0.045435306
C	-2.525619174	-0.447098442	-0.045435297
C	1.251374112	0.219847066	-0.072717078
C	-2.655768149	-1.861552644	0.045096119
H	-1.778906434	-2.497435813	0.095587918
C	0.000000042	-0.467794339	0.037363844
H	0.000000088	-1.535560466	0.194741247
C	-3.713451274	0.331369370	-0.119423272
H	-3.626086989	1.409258028	-0.187684917

C	2.655768106	-1.861552559	0.045096373
H	1.778906339	-2.497435643	0.095588379
C	3.713451359	0.331369374	-0.119423500
H	3.626087137	1.409258017	-0.187685338
C	-4.957919099	-0.275379309	-0.098101553
H	-5.854181171	0.335772791	-0.151967001
C	-3.908750435	-2.455979310	0.066474748
H	-3.986877339	-3.536834023	0.137103983
C	-5.068242834	-1.672590687	-0.004469789
H	-6.046639708	-2.143063780	0.013005465
C	5.068242808	-1.672590751	-0.004469736
H	6.046639655	-2.143063896	0.013005542
C	3.908750356	-2.455979289	0.066475030
H	3.986877201	-3.536833991	0.137104472
C	4.957919146	-0.275379377	-0.098101753
H	5.854181224	0.335772656	-0.151967376
B	0.000000010	2.340323273	0.067593951

Cartesian coordinates of the QM regions of QM/MM optimized structures (unit: angstrom).

monomer.S_0

F	1.40435800	-2.76873000	1.74396300
O	0.00184600	-1.73587200	0.18365300
F	1.39627400	-3.50323000	-0.42526300
O	2.45404300	-1.48700500	0.09164200
C	-1.48752500	0.04632100	-0.20709900
C	3.52219100	0.56413900	-0.36470500
C	-0.11181600	-0.46699800	-0.09565000
C	2.29145400	-0.22136700	-0.17682200
C	1.01524300	0.33249800	-0.28135200
H	0.90070300	1.37131000	-0.53275500
C	3.48609600	1.96607700	-0.49750500
H	2.54575800	2.50367400	-0.46354000
C	4.76627300	-0.09236700	-0.38286900
H	4.79998000	-1.17003800	-0.26973900
C	5.94324900	0.63579900	-0.52858700
H	6.89311300	0.11127800	-0.53540900
C	-2.56768100	-0.85170600	-0.14347600
H	-2.36894500	-1.91102600	-0.03040500
C	5.89583800	2.02707400	-0.65337800
H	6.80852100	2.60479300	-0.75049400
C	-3.87537800	-0.38218500	-0.21158500
H	-4.70255400	-1.07865800	-0.16694700
C	4.66505800	2.68761000	-0.64083800
H	4.62399600	3.76780500	-0.74191700
C	-1.75062900	1.42241800	-0.34637100

H	-0.94140800	2.13920500	-0.38979800
C	-3.05929800	1.88774200	-0.39165700
H	-3.25240100	2.95341100	-0.45997700
C	-4.12289000	0.98708300	-0.32367400
H	-5.14009700	1.35842700	-0.34597600
B	1.32079500	-2.41907300	0.41568700

monomer.S_1

F	1.37279600	-2.61780200	1.73621300
O	-0.00887800	-1.84633700	0.00563400
F	1.39595000	-3.66970600	-0.30234000
O	2.45069600	-1.60924500	-0.08906700
C	-1.47715600	-0.00235600	-0.22386300
C	3.51803600	0.48833300	-0.37994400
C	-0.14552000	-0.54359600	-0.13717500
C	2.32029200	-0.30373600	-0.22298500
C	1.02213400	0.26914500	-0.22733300
H	0.91573700	1.33340500	-0.33154200
C	3.47270000	1.90917000	-0.53396700
H	2.52475700	2.43443000	-0.54539300
C	4.78882600	-0.14671700	-0.37186700
H	4.83634900	-1.22274300	-0.25003800
C	5.94779100	0.60104700	-0.49561500
H	6.90771400	0.09527300	-0.47674200
C	-2.60038100	-0.86919500	-0.13734800
H	-2.43101300	-1.93272000	-0.01799200
C	5.88528700	2.00117400	-0.63359000
H	6.79236800	2.59016600	-0.71396000
C	-3.88564600	-0.35779200	-0.18392200
H	-4.73470500	-1.02631000	-0.11398900
C	4.64053000	2.64246300	-0.65673900
H	4.58607100	3.72063900	-0.77484000
C	-1.71676400	1.39804900	-0.38217600
H	-0.89063800	2.09269400	-0.46363200
C	-3.00852700	1.89497900	-0.40887500
H	-3.17252900	2.96460200	-0.49412100
C	-4.10088900	1.02656900	-0.30754500
H	-5.10778200	1.42601100	-0.31197600
B	1.30956900	-2.47253700	0.36368200

monomer.T_1

F	1.37095400	-2.60790700	1.73338200
O	-0.01035000	-1.84846500	-0.00267800
F	1.39796100	-3.66815600	-0.30215300
O	2.45229100	-1.60890900	-0.09484400
C	-1.48084000	0.00663700	-0.22194100

C	3.51985500	0.49867500	-0.37838500
C	-0.15457500	-0.54132200	-0.13927600
C	2.33301700	-0.29856600	-0.22442800
C	1.02120900	0.26561300	-0.23414300
H	0.91389400	1.33104100	-0.34596400
C	3.47573300	1.91473500	-0.53049900
H	2.53055400	2.44498000	-0.54116100
C	4.79167800	-0.13591500	-0.37167400
H	4.83922500	-1.21207400	-0.25127600
C	5.95141900	0.61010500	-0.49551300
H	6.90994400	0.10182500	-0.47732100
C	-2.60182400	-0.86223000	-0.13683800
H	-2.42969500	-1.92554300	-0.01991100
C	5.88978500	2.00712600	-0.63182600
H	6.79595600	2.59689200	-0.71242900
C	-3.88913900	-0.35560000	-0.18286700
H	-4.73503100	-1.02802900	-0.11433200
C	4.64475600	2.64735800	-0.65272900
H	4.58981900	3.72556600	-0.76911000
C	-1.72308800	1.40150600	-0.37645000
H	-0.90166500	2.10183600	-0.45593000
C	-3.01675000	1.89552300	-0.40187500
H	-3.18252200	2.96493200	-0.48394400
C	-4.10742000	1.02541300	-0.30345900
H	-5.11447900	1.42344100	-0.30752800
B	1.30890300	-2.47105300	0.36101300

dimer.S_0

O	2.76164300	-1.58987700	-1.41494500
O	0.30169600	-1.67881000	-1.30796100
F	1.54829800	-3.27577400	-2.47726300
F	1.63706900	-3.23009100	-0.18565800
C	2.68928300	-0.28714800	-1.39469700
C	-1.05547100	0.24314600	-1.17354700
C	3.97556200	0.42575500	-1.43629700
C	0.27850100	-0.37304500	-1.27257000
C	4.03647100	1.81552500	-1.65214000
H	3.13238300	2.38725600	-1.82332300
C	1.45940800	0.36512600	-1.31836000
H	1.42771600	1.43665400	-1.24559000
C	5.17221600	-0.29378700	-1.26255100
H	5.12992900	-1.36562200	-1.10495500
C	-1.22850500	1.63987000	-1.21478500
H	-0.37806700	2.29879800	-1.33670900
C	-2.18672600	-0.57937800	-1.02060800
H	-2.05654700	-1.65484000	-0.98873000

C	6.39886400	0.36293600	-1.29997100
H	7.31205200	-0.20753400	-1.16548700
C	5.26402700	2.46511000	-1.68848400
H	5.30199800	3.53695200	-1.85447800
C	6.44625400	1.74299300	-1.51398700
H	7.39644200	2.26297800	-1.56183300
C	-3.61021900	1.36891800	-0.95233200
H	-4.59285200	1.81065600	-0.84090200
C	-2.49687900	2.19760600	-1.10259700
H	-2.61339600	3.27532100	-1.08779300
C	-3.45586100	-0.01878200	-0.91254600
H	-4.32083000	-0.66010900	-0.78238400
B	1.56473800	-2.48630400	-1.34828000
O	-3.64429100	3.06038900	2.35614700
O	-1.18283800	3.11924100	2.25716700
F	-2.42096200	4.79386000	3.32048100
F	-2.49732100	4.61228100	1.03484900
C	-3.58698100	1.75719100	2.36205400
C	0.15246100	1.18017800	2.15414400
C	-4.88068600	1.06011100	2.42813200
C	-1.17514400	1.81299400	2.23943000
C	-4.95589200	-0.32541000	2.66508700
H	-4.05731300	-0.90489200	2.83856500
C	-2.36472400	1.08989400	2.28923400
H	-2.34606300	0.01709100	2.23923200
C	-6.07059600	1.79111100	2.25519400
H	-6.01685400	2.86005500	2.08240700
C	0.30571400	-0.21931200	2.16105300
H	-0.55563900	-0.86845500	2.25425900
C	1.29733400	1.98997300	2.03999100
H	1.18286400	3.06785000	2.03781700
C	-7.30443400	1.14976400	2.31357200
H	-8.21248800	1.72911000	2.18121500
C	-6.19054100	-0.95980400	2.72077200
H	-6.23959500	-2.02846700	2.90291700
C	-7.36590500	-0.22660700	2.54689100
H	-8.32133600	-0.73461600	2.61255200
C	2.69439800	0.02400200	1.93352100
H	3.67114500	-0.43054800	1.82262600
C	1.56699500	-0.79197900	2.04551400
H	1.66839900	-1.87083200	1.99970900
C	2.56031700	1.41455900	1.93456900
H	3.43529000	2.04944800	1.83957100
B	-2.43792700	3.93981800	2.24295900

dimer.S_1

O	2.83085400	-1.64109400	-1.41954400
O	0.35990800	-1.74216100	-1.34426200
F	1.63312700	-3.35569900	-2.46197900
F	1.69075700	-3.25444200	-0.17074200
C	2.75909800	-0.31765000	-1.43181500
C	-0.97598400	0.20022400	-1.13260600
C	4.02944700	0.40346500	-1.47022000
C	0.33470200	-0.41070400	-1.31495300
C	4.08555500	1.80061600	-1.67564500
H	3.17860900	2.36500900	-1.85957700
C	1.51594200	0.32191000	-1.38642900
H	1.48135100	1.39603700	-1.34247700
C	5.24104100	-0.29732700	-1.29289300
H	5.21207700	-1.37218600	-1.15294900
C	-1.16605000	1.60746400	-1.12723700
H	-0.33246300	2.26881000	-1.32864900
C	-2.10922800	-0.62409100	-0.91173700
H	-1.97810300	-1.69930700	-0.93705500
C	6.45845100	0.37703300	-1.31021200
H	7.37573800	-0.18691100	-1.17431500
C	5.30416200	2.46470700	-1.68919100
H	5.32838700	3.53870700	-1.84926800
C	6.49706300	1.76022300	-1.50619600
H	7.43972400	2.29371400	-1.53904900
C	-3.51546800	1.32980200	-0.62192400
H	-4.49228300	1.76346100	-0.43434800
C	-2.41364200	2.15933000	-0.88340500
H	-2.53136000	3.23761700	-0.85200500
C	-3.35442500	-0.06886000	-0.66217300
H	-4.20916400	-0.71460400	-0.48366000
B	1.62895000	-2.51653900	-1.36061900
O	-3.72078700	3.09899400	2.45002900
O	-1.25465100	3.18659400	2.32568400
F	-2.49598100	4.80039300	3.46530400
F	-2.60166300	4.70877400	1.17502900
C	-3.67639600	1.80537300	2.38691800
C	0.07278900	1.26067100	2.06491300
C	-4.94262800	1.08833600	2.46299200
C	-1.22260800	1.88526400	2.21973100
C	-4.98847700	-0.30894400	2.66110800
H	-4.07857500	-0.87970100	2.80206500
C	-2.42614800	1.14358800	2.25541500
H	-2.40439000	0.07592900	2.14244800
C	-6.14904700	1.80773900	2.32775600
H	-6.11510700	2.88135600	2.18084600
C	0.22666700	-0.15508400	1.98205100

H	-0.63119700	-0.80715600	2.07616700
C	1.22847700	2.08336800	1.95066800
H	1.11359000	3.15811300	2.02072200
C	-7.36665400	1.14283800	2.37789400
H	-8.28568100	1.70811700	2.26831000
C	-6.21042700	-0.96309200	2.71646700
H	-6.24093500	-2.03645100	2.86883000
C	-7.39948000	-0.24304500	2.57396900
H	-8.34521800	-0.76867800	2.63289900
C	2.59022300	0.11370700	1.61224400
H	3.55872400	-0.32338200	1.39814700
C	1.47087400	-0.71651100	1.76541900
H	1.57049600	-1.78646300	1.62588500
C	2.46597800	1.51797500	1.72812200
H	3.34128200	2.14662200	1.60433900
B	-2.51197200	4.00266000	2.35260200

dimer.T_1

O	2.82227200	-1.63182000	-1.46237100
O	0.36369700	-1.75151700	-1.35778900
F	1.62759800	-3.32482800	-2.53899700
F	1.71456400	-3.29372900	-0.24684600
C	2.73476000	-0.32900700	-1.43630500
C	-1.00908900	0.15018700	-1.14702000
C	4.01153400	0.40041400	-1.46316000
C	0.32645300	-0.44512300	-1.30428100
C	4.05636100	1.79238200	-1.67056500
H	3.14650900	2.35373400	-1.84639700
C	1.49630900	0.30837000	-1.36393300
H	1.45226100	1.37866200	-1.27613700
C	5.21566100	-0.30462900	-1.28223300
H	5.18595200	-1.37787100	-1.13145300
C	-1.20612100	1.54446000	-1.15663200
H	-0.37614200	2.21765000	-1.33177600
C	-2.11838700	-0.69252100	-0.93297600
H	-1.96874400	-1.76552900	-0.92797300
C	6.43359800	0.36839500	-1.30406700
H	7.35207200	-0.19168600	-1.16351300
C	5.27567900	2.45784300	-1.69198200
H	5.30134900	3.53099900	-1.85196300
C	6.46546500	1.75004700	-1.51017900
H	7.40918500	2.28235100	-1.54634100
C	-3.55879500	1.23437100	-0.71016400
H	-4.53947100	1.65589200	-0.51757200
C	-2.46979000	2.08209300	-0.93901500
H	-2.60066700	3.15727900	-0.89763800
C	-3.38174700	-0.15528600	-0.71801900

H	-4.22726000	-0.80893000	-0.53455500
B	1.63587600	-2.54190700	-1.40588900
O	-3.70046800	3.12529400	2.40800800
O	-1.21978000	3.19047800	2.35288200
F	-2.49053800	4.88914500	3.31367700
F	-2.52577900	4.60687800	1.03333700
C	-3.67133300	1.80864800	2.39881600
C	0.09940000	1.23040500	2.12344600
C	-4.92456000	1.09577600	2.45910900
C	-1.17819400	1.87144700	2.24994100
C	-4.98305800	-0.30982400	2.64433200
H	-4.07700000	-0.88649500	2.78873100
C	-2.41117100	1.14862100	2.28711600
H	-2.39378700	0.07545000	2.20169400
C	-6.14017900	1.81250300	2.32604900
H	-6.10536300	2.88772600	2.19223000
C	0.24594100	-0.18620200	2.06164500
H	-0.62030100	-0.83222200	2.12862600
C	1.27548300	2.02772000	2.03733500
H	1.17659600	3.10581400	2.08545400
C	-7.35620400	1.14772800	2.36912000
H	-8.27444700	1.71630300	2.26468800
C	-6.20541200	-0.96123900	2.68883400
H	-6.23572300	-2.03623100	2.83388200
C	-7.39684300	-0.24183400	2.55203700
H	-8.34353400	-0.76584700	2.60761000
C	2.63936600	0.03882400	1.83187900
H	3.61198000	-0.42023500	1.69792200
C	1.49661300	-0.76514300	1.91772400
H	1.58409600	-1.84220700	1.82053400
C	2.51920300	1.43865400	1.89690800
H	3.40441000	2.06266600	1.82295600
B	-2.48101300	3.98923300	2.27487700

References

(1) Wang, J.-X.; Niu, L.-Y.; Chen, P.-Z.; Chen, Y.-Z.; Yang, Q.-Z.; Boulatov, R. Ratiometric O_2 Sensing based on Selective Self-Sensitized Photooxidation of Donor-Acceptor Fluorophores. *Chem. Commun.* **2019**, *55*, 7017-7020.

(2) Wang, J.-X.; Yu, Y.-S.; Niu, L.-Y.; Zou, B.; Wang, K.; Yang, Q.-Z. A Difluoroboron Beta-Diketonate based Thermometer with Temperature-Dependent Emission Wavelength. *Chem. Commun.* **2020**, *56*, 6269-6272.

(3) Vosko, S. H.; Wilk, L.; Nusair, M. Accurate Spin-Dependent Electron Liquid Correlation Energies for Local Spin Density Calculations: A Critical Analysis. *J. Phys.* **1980**, *58*, 1200-1211.

(4) Becke, A. D. Density-Functional Exchange-Energy Approximation with Correct

Asymptotic Behavior. *Phys. Rev. A.* **1988**, *38*, 3098-3100.

(5) Lee, C.; Yang, W.; Parr, R. G. Development of the Colle-Salvetti Correlation-Energy Formula into a Functional of the Electron Density. *Phys. Rev. B.* **1988**, *37*, 785.

(6) Ditchfield, R.; Hehre, W. J.; Pople, J. A. Self-Consistent Molecular-Orbital Methods. IX. An Extended Gaussian-Type Basis for Molecular-Orbital Studies of Organic Molecules. *J. Chem. Phys.* **1971**, *54*, 724-728.

(7) Hariharan, P. C.; Pople, J. A. The Influence of Polarization Functions on Molecular Orbital Hydrogenation Energies. *Theor. Chim. Acta.* **1973**, *28*, 213-222.

(8) Barone, V.; Cossi, M. Quantum Calculation of Molecular Energies and Energy Gradients in Solution by a Conductor Solvent Model. *J. Phys. Chem. A.* **1998**, *102*, 1995-2001.

(9) Tomasi, J.; Mennucci, B.; Cammi, R. Quantum Mechanical Continuum Solvation *Models. Chem. Rev.* **2005**, *105*, 2999-3093.

(10) Maseras, F.; Morokuma, K. IMOMM: A New Integrated Ab Initio + Molecular Mechanics Geometry Optimization Scheme of Equilibrium Structures and Transition States. *J. Comput. Chem.* **1995**, *16*, 1170.

(11) Vreven, T.; Morokuma, K.; Farkas, O¨.; Schlegel, H. B.; Frisch, M. J. Geometry Optimization with QM/MM, ONIOM, and Other Combined Methods. I. Microiterations and Constraints. *J. Comput. Chem.* **2003**, *24*, 760.

(12) Rappé, A. K.; Casewit, C. J.; Colwell, K. S.; Goddard, W. A., III; Skiff, W. M. UFF, a Full Periodic Table Force Field for Molecular Mechanics and Molecular Dynamics Simulations. *J. Am. Chem. Soc.* **1992**, *114*, 10024.

(13) Rappé, A. K.; Goddard, W. A., III. Charge Equilibration for Molecular Dynamics Simulations. *J. Phys. Chem.* **1991**, *95*, 3358.

(14) Frisch, M. J.; Trucks, G. W.; Schlegel, H. B.; Scuseria, G. E.; Robb, M. A.; Cheeseman, J. R.; Scalmani, G.; Barone, V.; Mennucci, B.; Petersson, G. A.; et al. *Gaussian 09, Revision D.01*, Gaussian, Inc., Wallingford CT, 2013.

(15) Frisch, M. J.; Trucks, G. W.; Schlegel, H. B.; Scuseria, G. E.; Robb, M. A.; Cheeseman, J. R.; Scalmani, G.; Barone, V.; Petersson, G. A.; Nakatsuji, H.; et al. *Gaussian 16, Revision A. 03*, Gaussian, Inc., Wallingford CT, 2016.

(16) Andersson, K.; Malmqvist, P.-Å.; Roos, B. O.; Sadlej, A. J.; Wolinski, K. Second-Order Perturbation Theory with a CASSCF Reference Function. *J. Phys. Chem. A.* **1990**, *94*, 5483-5488.

(17) Andersson, K.; Malmqvist, P.-Å.; Roos, B. O. Second-Order Perturbation Theory with a Complete Active Space Self-Consistent Field Reference Function. *J. Chem. Phys.* **1992**, *96*, 1218-1226.

(18) Roos, B. O.; Lindh, R.; Malmqvist, P.-Å.; Veryazov, V.; Widmark, P.- O. Main Group Atoms and Dimers Studied with a New Relativistic ANO Basis Set, *J. Phys. Chem. A.* **2004**, *108*, 2851-2858.

(19) Aquilante, F.; Lindh, R.; Perdersen, T. B. Unbiased Auxiliary Basis Sets for Accurate Two-Electron Integral Approximations. *J. Chem. Phys.* **2007**, *127*, 114107.

(20) Forsberg, N.; Malmqvist, P.-Å. Multiconfiguration Perturbation Theory with Imaginary Level Shift. *Chem. Phys. Lett.* **1997**, *274*, 196-204.

(21) Ghigo, G.; Roos, B. O.; Malmqvist, P.-Å. A Modified Definition of the Zeroth-Order Hamiltonian in Multiconfigurational Perturbation Theory (CASPT2). *Chem. Phys. Lett.* **2004**, *396*, 142-149.

(22) Miertuš, S.; Scrocco, E.; Tomasi, J. Electrostatic Interaction of a Solute with a Continuum. A Direct Utilizaion of AB Initio Molecular Potentials for the Prevision of Solvent Effects. *Chem. Phys.* **1981**, *55*, 117-129.

(23) Cammi, R.; Thomasi, J. Analytical Derivatives for Molecular Solutes. I. Hartree–Fock Energy First Derivatives with Respect to External Parameters in the Polarizable Continuum Model. *J. Chem. Phys.* **1994**, *100*, 7495-7502.

(24) Karlström, G.; Lindh, R.; Malmqvist, P.-Å.; Roos, B. O.; Ryde, U.; Veryazov, V.; Widmark, P.-O.; Cossi, M.; Schimmelpfennig, B.; Neogrady, P.; et al. MOLCAS: a Program Package for Computational Chemistry. *Comput. Mater. Sci.* **2003**, *28*, 222-239.

(25) Aquilante, F.; Autschbach, J.; Carlson, R. K.; Chibotaru, L. F.; Delcey, M. G.; De Vico, L.; Fdez. Galván, I.; Ferré, N.; Frutos, L. M.; Gagliardi, L.; et al. MOLCAS 8: New Capabilities for Multiconfigurational Quantum Chemical Calculations across the Periodic Table: Molcas 8. *J. Comput. Chem.* **2016**, *37*, 506-541.
